# Understanding the relationship between loneliness, substance use traits and psychiatric disorders: A genetically informed approach

**DOI:** 10.1016/j.psychres.2023.115218

**Published:** 2023-07

**Authors:** Ellen Martin, Tabea Schoeler, Jean-Baptiste Pingault, Wikus Barkhuizen

**Affiliations:** a Division of Psychology and Language Sciences, University College London, London, United Kingdom; b Department of Computational Biology, University of Lausanne, Lausanne, Switzerland; c Social, Genetic and Developmental Psychiatry Centre, Institute of Psychiatry, King's College, London, United Kingdom

**Keywords:** Genetics, Mendelian randomization, Genomic structural equation modelling, Depression, Neurodevelopmental disorders, Mood disorders

## Abstract

Loneliness is a common, yet distressing experience associated with adverse outcomes including substance use problems and psychiatric disorders. To what extent these associations reflect genetic correlations and causal relationships is currently unclear. We applied Genomic Structural Equation Modelling (GSEM) to dissect the genetic architecture between loneliness and psychiatric-behavioural traits. Included were summary statistics from 12 genome-wide association analyses, including loneliness and 11 psychiatric phenotypes (range *N*: 9,537 – 807,553). We first modelled latent genetic factors amongst the psychiatric traits to then investigate potential causal effects between loneliness and the identified latent factors, using multivariate genome-wide association analyses and bidirectional Mendelian randomization. We identified three latent genetic factors, encompassing neurodevelopmental/mood conditions, substance use traits and disorders with psychotic features. GSEM provided evidence of a unique association between loneliness and the neurodevelopmental/mood conditions latent factor. Mendelian randomization results were suggestive of bidirectional causal effects between loneliness and the neurodevelopmental/mood conditions factor. These results imply that a genetic predisposition to loneliness may elevate the risk of neurodevelopmental/mood conditions, and vice versa. However, results may reflect the difficulty of distiguishing between loneliness and neurodevelopmental/mood conditions, which present in similar ways. We suggest, overall, the importance of addressing loneliness in mental health prevention and policy.

## Highlights

• Genomic Structural Equation Modelling was used to implement factor analysis on 11 psychiatric traits.
• The 11 psychiatric traits clustered into three latent genetic factors encompassing neurodevelopmental/mood conditions, substance use traits and disorders with psychotic features.
• Loneliness was uniquely genetically associated with the latent factor representing neurodevelopmental/mood conditions.
• Mendelian randomization provided evidence suggesting a bidirectional causal effect between loneliness and neurodevelopmental/mood conditions.

## 1 Introduction

Loneliness is a painful, distressing experience that occurs across cultures and demographic categories, throughout the lifespan (Barreto et al., 2021; Surkalim et al., 2022). Distinct from objectively being alone (i.e., isolation), loneliness refers to the subjective, pervasive feeling of isolation (Cacioppo and Cacioppo, 2018). Although the experience of loneliness is temporary for many, approximately 15% to 30% of the general population experience prolonged periods of loneliness (Hawkley and Cacioppo, 2010). There is increasing recognition of loneliness as a significant public health concern (Fried et al., 2020; Leigh-Hunt et al., 2017; Rodney et al., 2021) because of its links to a range of adverse mental and physical health outcomes (Ingram et al., 2020b; Leathem et al., 2021; Lim et al., 2018; Wang et al., 2018).

Understanding the causes and consequences of loneliness is, however, challenging. While loneliness has been described as a risk factor for poor mental health and behavioural problems (e.g., substance use problems) (Cacioppo et al., 2015; Copeland et al., 2018; Erzen and Çikrikci, 2018; Houghton et al., 2020; Ingram et al., 2020a; Matthews et al., 2019; Wang et al., 2020), poor mental health and behavioural problems may also reciprocally contribute to experiences of loneliness (Cacioppo et al., 2015; Ingram et al., 2020b, 2020a; Leathem et al., 2021; Vogel et al., 2013; Wootton et al., 2020). Individuals with mood problems, neurodevelopmental conditions, disorders with psychotic features and those who heavily use substances are especially vulnerable to experiences of loneliness (Giacco, 2023; Giacco et al., 2016; Ingram et al., 2020a). Because of this reverse causality, the directionality of effects between loneliness and mental health remains unclear. In addition, substantial genetic correlations between loneliness and a number of psychiatric disorders and substance use traits have been reported (Gao et al., 2016; Jang et al., 2022; Matthews et al., 2019). For example, more than half of 61 assessed physical and mental health traits were found to be genetically correlated with loneliness (Abdellaoui et al., 2019), most of which related to psychiatric outcomes and substance use. As such, the observed correlations between loneliness and psychiatric phenotypes may be explained by a shared genetic liability.

To better disentangle causes, consequences and comorbidities, multivariate genomic approaches combining structural modelling with genetically informed causal inference methods are increasingly applied. One such multivariate genetic method is Genomic Structural Equation Modelling (GSEM) (Grotzinger et al., 2019). GSEM relies on summary data from previous Genome-Wide Association Studies (GWAS) to jointly model multiple genetic correlations between phenotypes and can thus adjust for genetic correlations between multiple phenotypes. Mendelian randomization (MR) (Richmond and Smith, 2022), a genetically informed causal inference method, can further enable the investigation of causal effects between phenotypes, such as loneliness and psychiatric phenotypes. Combining these two genetically informed approaches to interrogate the complex co-occurrence of loneliness and psychiatric phenotypes may help to address the gaps in our understanding of loneliness.

In summary, loneliness is intricately intertwined with various psychiatric and substance-related phenotypes. In this study, we aim to disentangle the associations between loneliness, substance use and psychiatric disorders, by combining GSEM and MR. Exploiting these two approaches allows us to scrutinise the shared and non-shared architecture between loneliness and psychiatric outcomes, and to examine causal questions concerning the aetiology of loneliness. In particular, we conduct two sets of complementary analyses:

1) We first conduct genomic factor analysis to determine how phenotypes indexing psychiatric outcomes and substance use cluster together. The selection of specific phenotypes was based on prior literature examining the latent ‘p-factor’ of common mental health conditions (Caspi et al., 2014) and the genetic architecture of mental health conditions (e.g., Abdellaoui et al., 2021; Lee et al., 2021). These studies suggest that certain psychiatric and substance use phenotypes differentially cluster alongside one another based on shared genetic liability. Based on the output of genomic factor analysis, we then investigate the degree to which the identified latent psychopathology factors associate with liability to loneliness. This is to evaluate the associations between the genetic liability underlying loneliness and the genetic liability underlying clusters of psychiatric phenotypes, rather than loneliness’ associations with individual, specific phenotypes.
2) We then examine whether genetic correlations between loneliness and these latent factors reflect causal processes. To this end, we conduct cross-trait GWAS for the latent genetic factors using GSEM. The resulting GWAS summary statistics are used in subsequent MR analyses to test possible bidirectional relationships between loneliness and the latent psychopathology factors.

## 2 Methods

Analyses were conducted in R version 4.0.3 (R Core Team, 2022). All analysis scripts are available at https://github.com/ellenmartin11/lone-GenSEM-MR.

### 2.1 Data selection and quality control

Publicly available summary statistics from GWAS conducted in individuals of European ancestry were selected based on criteria recommended by Choi et al. (2020) to ensure adequate statistical power. This included selecting the largest and most recent GWAS available, with single nucleotide polymorphism (SNP) heritability of at least 5% and an associated SNP heritability *z*-score of at least two. In our study, we selected summary statistics for loneliness, measured using self-report responses to the UCLA Loneliness Scale (Day et al., 2018), clinical diagnoses of ADHD (Demontis et al., 2018), anxiety (Otowa et al., 2016), autism (Grove et al., 2019), bipolar disorder (Stahl et al., 2019), major depression (Howard et al., 2019), PTSD (Nievergelt et al., 2019), and schizophrenia (Pardiñas et al., 2018), the amount of alcohol consumed per day in grams (Schumann et al., 2016), clinical diagnosis of cannabis use disorder (Johnson et al., 2020) and lifetime smoking (Wootton et al., 2020), measured as a combined index of current smoking status, duration of smoking and smoking frequency. Table 1 provides details of the summary statistics used (average GWAS *N* = 227,089, range 9537 - 807,553). Further details of the summary statistics used and of how phenotypes were measured can be found in the Supplementary Material (Supplementary Table 1).

**Table 1:** Summary Statistics of the 11 Phenotypes Included.

GWAS	N	Source	SE	N SNPs	SNP-h^2^	Measure	Power
ADHD	55,374	Demontis et al. (2018)	0.015	579,907	0.236	binary	15.6
Alcohol Consumption	70,460	Schumann (2015)	0.008	1065,953	0.050	continuous	6.3
Anxiety	15,730	Otowa et al. (2016)	0.030	1100,798	0.079	binary	2.6
Autism	46,350	Grove et al. (2019)	0.010	1048,195	0.118	binary	11.8
Bipolar Disorder	51,710	Stahl et al. (2019)	0.008	1097,032	0.170	binary	22.6
Cannabis Use Disorder	364,701	Johnson et al. (2020)	0.006	1170,813	0.070	binary	11.7
Loneliness	445,024	Day et al. (2018)	0.002	1179,978	0.042	continuous	21.9
Major Depression	807,553	Howard et al. (2019)	0.003	1161,617	0.089	binary	29.7
PTSD	9537	Nievergelt et al. (2019)	NA	1170,304	0.050	binary	NA
Smoking	462,690	Wooton et al. (2019)	0.003	1173,173	0.090	continuous	29.5
Schizophrenia	105,318	Pardiñas et al. (2018)	0.014	1151,269	0.412	binary	30.3

*Note.* For binary phenotypes, the sample size (N) refers to the Effective Sample Size. *N* = effective sample size; GWAS = Genome-Wide Association Study; SE = standard error, N SNPs = number of SNPs; SNP-*h^2^ =* single-nucleotide polymorphism heritability index.

We applied standard quality control filters (imputation quality > 90%, minor allele frequency > 1%) for SNP selection. Additional details of quality control can be found in the Supplementary Material.

### 2.2 Factor analysis using genomic structural equation modelling

To first model the genetic architecture underlying the substance use and psychiatric phenotypes, we used *GenomicSEM* version 0.0.2 (Grotzinger et al., 2019). GSEM uses Linkage-Disequilibrium Score Regression (LDSC) (Bulik-Sullivan et al., 2015) to compute unadjusted genetic covariances between the specified phenotypes. The LDSC genetic covariance matrices were used for Principal Component Analysis (PCA), Exploratory Factor Analysis (EFA) and Confirmatory Factor Analysis (CFA). To guide our decision on the number of factors to retain, we used PCA to evaluate eigenvalues and generate a scree plot. Kaiser's Criterion recommends retaining components with eigenvalues that surpass one and the plateau of the scree plot indicates the number of components to retain. To prevent model over-fitting, PCA and EFA were conducted on odd chromosomes whilst CFA was performed on even chromosomes. The best-fitting model, as indicated by the lowest Standardised Root Mean Square Residual (SRMR) and highest Comparative Fit Index (CFI) values, was retained and used as the basis of subsequent GSEM models with loneliness.

We specified three GSEM models regressing loneliness and the latent genetic factors for psychopathology. In all models, loneliness was constructed as a latent factor (LONE) due to syntax requirements of *lavaan* (Rosseel, 2012) for the genomic structural equation model to converge. In the first model, separate associations between loneliness and the latent factors were modelled. This model is unadjusted since it does not correct for the intercorrelations between the latent genetic factors. We then specified a second, adjusted multivariate model controlling for genetic correlation by regressing loneliness onto all the latent factors simultaneously and specifying intercorrelations between the latent factors. The regression coefficient estimates from this model are therefore partial genetic correlations between loneliness and the latent factors that are statistically adjusted to account for the intercorrelations between the latent factors. The third model was the same as the second, except we applied constraints to check for potential over-inflation of standardised coefficients and improve parsimony. The path diagram for the final model is shown in Fig. 2 of the results section. Alternate model specifications can be found in the Supplementary Material alongside their path diagrams.

### 2.3 Bidirectional MR analyses

Mendelian randomization (MR) is a causal inference method leveraging the results obtained from genome-wide analyses. MR relies on the use of genetic instruments to estimate the causal effect of an exposure on an outcome. If valid instruments are used, MR estimates are free from biases due to the residual confounding that affects most estimates obtained from observational data. A valid genetic instrument must (1) be robustly associated with the exposure, (2) not be associated with any factor that confounds the relationship between the exposure and the outcome, and (3) act on the outcome only through the exposure (i.e., not due to horizontal pleiotropy) (Burgess et al., 2017).

To derive instruments for MR, we conducted a multivariate GWAS on the latent factors as modelled in the third structural model described above. The resulting SNP estimates on the latent factors were then used as the input for bidirectional MR analyses, testing for causality between loneliness and the latent factors. We used the TwoSampleMR package (Hemani et al., 2017) in R, including LD-independent (10,000 kb clump window, r^2^ < 0.001) genome-wide significant variants (*p* < 5 × 10^−08^) as the genetic instruments. Inverse-Variance Weighted (IVW) MR (Burgess et al., 2013) was used to obtain the exposure-outcome estimate. We evaluated heterogeneity using Cochran's *Q* and checked for horizontal pleiotropy using the MR-Egger intercept term. Given the nature of our study, focusing on latent genetic factors representing multiple traits, we conducted additional sensitivity checks to evaluate potential violations of MR assumption (2). First, when selecting genetic instruments for the latent genetic factor, we filtered variants according to the heterogeneity chi-square statistic, Q_SNP_ (Grotzinger et al., 2019). Variants with Q_SNP_ *p* < 5 × 10^−08^ were excluded, indexing SNPs that do not act predominantly through the latent factor. Second, as horizontal pleiotropy can confound the association between the exposure and the outcome, we implemented several MR sensitivity analyses, including MR-Egger (Bowden et al., 2015), Weighted Median (Bowden et al., 2016) and Weighted Mode MR (Hartwig et al., 2017). MR Steiger (Hemani et al., 2017) was used to ensure that the genetic instruments used in MR analyses operated in the correct causal direction, i.e., from exposure to outcome. This was implemented by retaining MR instruments with a greater exposure *r^2^* than outcome *r^2^*, indicating that they likely operate in the correct causal direction. MR was then conducted using only these instruments.

Using a small number of instruments in MR analyses risks biasing estimated causal effects. Therefore, we conducted additional sensitivity analyses specifying a less stringent *p*-value threshold (*p* < 5 × 10^−07^) to increase the number of instruments. Due to the potential inclusion of weaker MR instruments when using a less stringent p-value threshold, we implemented MR Robust Adjusted Profile Score (RAPS) (Zhao et al., 2018). Details pertaining to all the MR methods and sensitivity analyses implemented can be found in the Supplementary Material.

## 3 Results

### 3.1 Latent genetic architecture

**Fig. 1:**
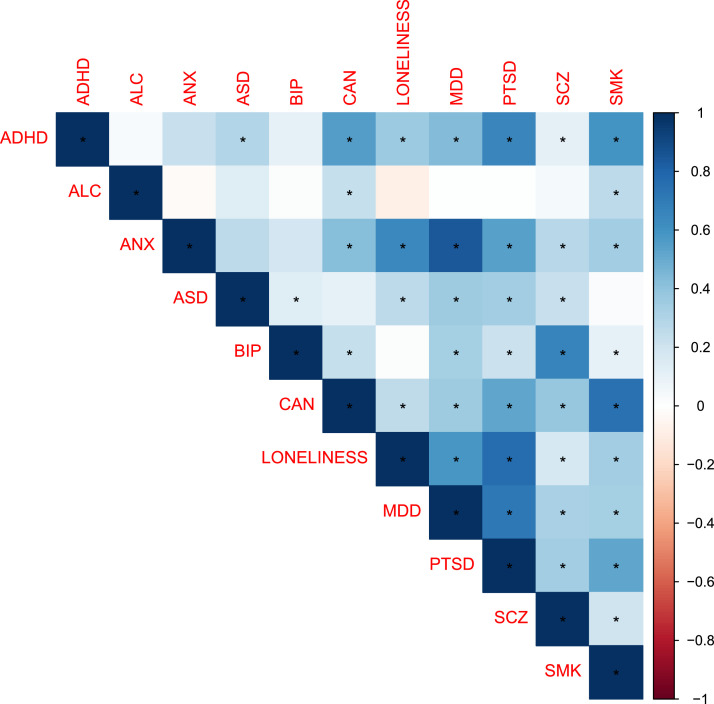
LDSC Correlation Heatmap of the 11 Included Phenotypes Note. Significant correlations between traits are represented with an asterisk (*). *P*-values have been corrected using the False-Discovery Ratio (FDR). ADHD = attention-deficit/hyperactivity disorder; ALC = alcohol consumption; ANX = anxiety; ASD = autism; BIP = bipolar disorder; CAN = cannabis use disorder; MDD = major depression; SCZ = schizophrenia; PTSD = post-traumatic stress disorder; SMK = smoking.

Fig. 1 shows the heatmap of bivariate genetic correlations obtained from LDSC regression. Loneliness was genetically correlated most strongly with PTSD (*r*(55) = 0.77, *p*_FDR_ = 5 × 10^−24^), anxiety (*r*(55) = 0.65, *p*_FDR_ = 6 × 10^−07^) and major depression (*r*(55) = 0.59, *p*_FDR_ = 5 × 10^−132^). Loneliness was also modestly correlated with autism (*r*(55) = 0.26, *p*_FDR_ = 8 × 10^10^), ADHD (*r*(55) = 0.36, *p*_FDR_ = 4 × 10^−15^), schizophrenia (*r*(55) = 0.18, *p*_FDR_ = 7 × 10^−15^), smoking (*r*(55) = 0.35, *p*_FDR_ = 2 × 10^−45^) and cannabis use disorder (*r*(55) = 0.26, *p*_FDR_ = 2 × 10^−10^).

The best-fitting factor analysis model was a three-factor solution accounting for 55.2% of the total variance across the ten phenotypes (*χ^2^*(33) = 139.42, *p* = 4.87×10^−15^, SRMR = 0.11, CFI = 0.93). Factors one, two and three each accounted for 27.5%, 13.9% and 13.9% of the variance respectively. Rejected factor structures and justification for their rejection can be found in the Supplementary Material alongside the PCA scree plot (Supplementary Fig. 1), EFA loadings and CFA output (Supplementary Tables 2*a* – 2*e*). Factors one, two and three were characterised as neurodevelopmental and mood conditions (NMD), substance use traits (SUT) and disorders with psychotic features (DPF), respectively.

The unadjusted genomic structural equation model provided evidence of positive associations between the latent genetic factor for loneliness (LONE) and all three latent factors. The path diagram for this model is available in the Supplementary Material (Supplementary Fig. 2*a).*

The results of the adjusted multivariate genomic structural equation model found the latent genetic factor for LONE to be positively and strongly associated with only NMD (β = 0.84, 95% CI = 0.536 – 1.138, *p* = 1.38×10^−16^). The loneliness factor did not appear to be associated with SUT (β = −0.05, 95% CI = −0.220 to −0.114, *p* = 3.45×10^−01^) nor with DPF (β = −0.18, 95% CI = −0.306 to −0.059, *p* = 1.18×10^−05^). This model's fit was reasonable (*χ^2^*(58) = 452.24, *p* = 1.96×10^−83^, SRMR = 0.09, CFI = 0.95). The three latent factors were genetically intercorrelated. The path diagram for this model is available in the Supplementary Material (Supplementary Fig. 2*b*).

**Fig. 2:**
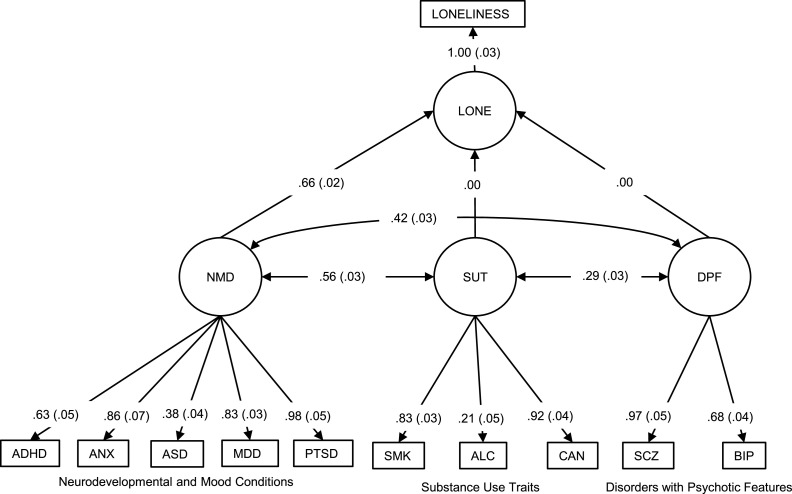
Adjusted and Constrained Multivariate Path Diagram Regressing Loneliness onto the Three Latent Factors, Accounting for Shared Genetic Covariation between the Latent Factors Note. Standard errors are shown in brackets alongside the beta coefficients. The single-headed arrows from the latent factors to loneliness represent that loneliness was regressed onto the three latent factors. ADHD = attention-deficit/hyperactivity disorder; ALC = alcohol consumption; ANX = anxiety disorder; ASD = autism; BIP = bipolar disorder; CAN = cannabis use disorder; MDD = major depression; SCZ = schizophrenia; PTSD = post-traumatic stress disorder; SMK = smoking. NMD = neurodevelopmental and mood conditions; SUT = substance use traits; DPF = disorders with psychotic features; LONE = latent loneliness factor.

The negative path loadings between the loneliness factor and SUT and DPF in the multivariate model may have arisen because of the reversal paradox (Tu et al., 2008), which can result from multicollinearity. To check whether these negative path loadings for SUT and DPF may have over-inflated the estimate between NMD and the loneliness factor, a third model was specified, and is shown in Fig. 2. This model constrained the paths between the loneliness factor and SUT and DPF to zero. NMD remained positively associated with the loneliness factor but with a lower effect size (β = 0.66, 95% CI = 0.560 - 0.761, *p* = 1.46×10^−65^). The fit was comparable to the unconstrained model (χ^2^(51) = 387.46, *p* = 5.99×10^−58^, SRMR = 0.10, CFI = 0.94). Since this model was more parsimonious and less likely to be over-inflated, it was used as the basis for the subsequent multivariate GWA and MR analyses. Full data pertaining to this model can be found in the Supplement (Supplementary Table 3).

GSEM model fit provided evidence of a strong genetic association between loneliness and the neurodevelopmental/mood conditions (NMD) latent factor. Evidence for associations between loneliness and the latent factors for disorders with psychotic features and for substance use traits was less strong. Therefore, we conducted a multivariate GWAS and follow-up MR analyses based on the NMD latent factor. A summary of the results of our GWAS of the NMD latent factor is available in the Supplementary Material (Supplementary Table 4, Supplementary Figs. 3*a* – 3*b).* The latent NMD factor was based on 1968,630 SNPs.

### 3.2 Mendelian randomization analyses

**Fig. 3:**
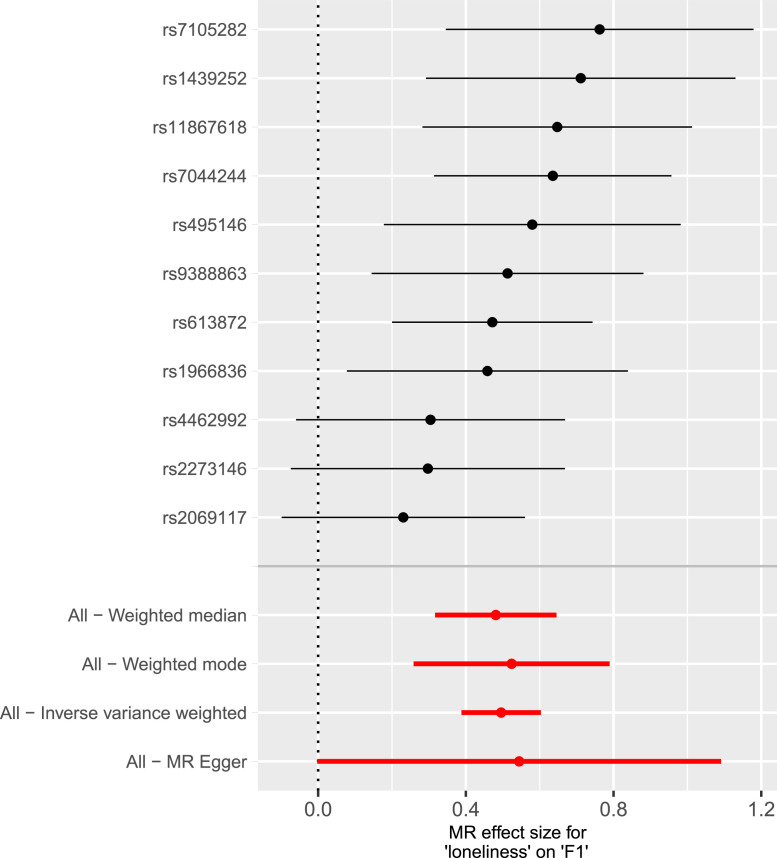
Mendelian Randomziation Forest Plot for Loneliness to Neurodevelopmental and Mood Conditions Note. The forest plot shows the effect sizes and confidence intervals of the 11 LD-independent SNPs used as MR instruments for loneliness to the neurodevelopmental and mood conditions latent factor. The four MR methods are shown a the bottom of the plot. F1 refers to the neurodevelopmental and mood conditions latent factor.

**Fig. 4:**
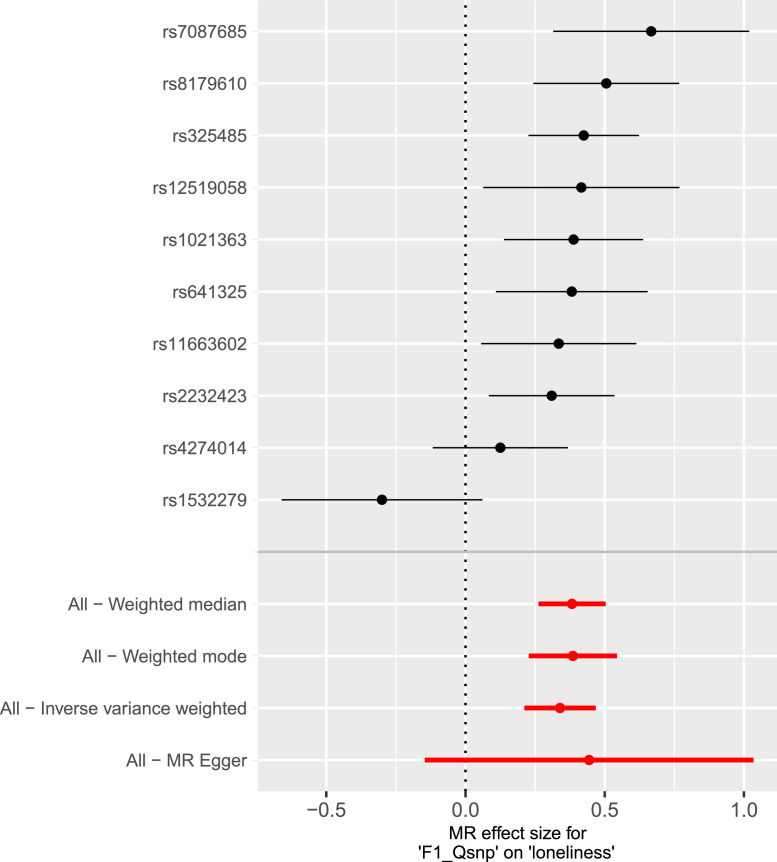
Mendelian Randomization Forest Plot for Neurodevelopmental and Mood Conditions to Loneliness. Note. The forest plot shows the effect sizes and confidence intervals of the 10 LD-independent SNPs used as MR instruments for the neurodevelopmental and mood conditions latent factor to the loneliness latent factor. The four MR methods are shown a the bottom of the plot. F1_Qsnp refers to the Q_SNP_ filtered neurodevelopmental and mood conditions latent factor.

The results of bidirectional MR shown in Table 2 suggest bidirectional causal effects between loneliness and neurodevelopmental/mood conditions. The main IVW estimate indicated a causal effect from loneliness to NMD (β_IVW_ = 0.496, 95% CI = 0.388 - 0.603, *p* = 1.49×10^−19^) using 11 genome-wide significant (*p* < 5 × 10^−8^) loneliness instruments. MR sensitivity analyses showed consistent results. The MR-Egger effect size was consistent with the main β_IVW_ estimate (β_Egger_ = 0.544, 95% CI = −0.002 - 1.091, *p* = 8.28×10^−02^). The I^2^ for MR-Egger was 0.972 from loneliness to NMD, suggesting instruments of adequate strength (Burgess and Thompson, 2017). In addition, there was no evidence of heterogeneity as indexed by Cochran's *Q* (*Q*_Egge_*_r_*(9) = 8.84, *p* = .452; *Q*_IVW_(10) = 8.87, *p* = .544) nor was there evidence of directional horizontal pleiotropy, according to the MR-Egger Intercept (Intercept = - 0.0007, *p* = .862)**.** Steiger filtering determined that all SNPs were operating in the correct direction. The forest plot in Fig. 3 shows the estimated causal effect of the 11 LD-independent instruments for MR analyses for loneliness to NMD. Additional MR analyses using 24 instruments and a p-value threshold of 5 × 10^−07^ (Supplementary Tables 5*i* – 5 m*,* Figs. 4d– 4e) demonstrated consistency with our main MR analyses.

**Table 2:** Table of Bidirectional Mendelian Randomization Results.

Exposure	Outcome	MR Method	*N* SNP	β (SE)	95% CI	*p*
Loneliness	NMD	Inverse Variance Weighted	11	0.496 (0.055)	0.388 - 0.603	1.49×10^−19^
Loneliness	NMD	MR Egger	11	0.544 (0.227)	−0.002 to 1.091	8.28×10^−02^
Loneliness	NMD	Weighted Mode	11	0.524 (0.055)	0.261 – 0.787	2.96×10^−03^
Loneliness	NMD	Weighted Median	11	0.481 (0.080)	0.324 – 0.639	2.16×10^−09^
NMD	Loneliness	Inverse Variance Weighted	10	0.339 (0.065)	0.211 – 0.468	2.16×10^−07^
NMD	Loneliness	MR Egger	10	0.444 (0.301)	−0.146 to 1.034	1.79×10^−01^
NMD	Loneliness	Weighted Mode	10	0.386 (0.088)	0.213 – 0.558	2.61×10^−03^
NMD	Loneliness	Weighted Median	10	0.383 (0.064)	0.257 – 0.508	3.60×10^−09^

*Note.* Loneliness and NMD are both continuous variables. NMD shown here is Q_SNP_ filtered. (MR = Mendelian randomization; *N* SNP = Number of Single-Nucleotide Polymorphisms; β = Beta; SE = Standard Error; CI = Confidence Interval; NMD = Neurodevelopmental and Mood Conditions).

Using 10 genome-wide significant NMD instruments, there was evidence of a causal effect from NMD to loneliness (β_IVW_ = 0.339, 95% CI = 0.211 - 0.468, *p* = 2.16×10^−07^). However, there was evidence of heterogeneity according to Cochran's *Q* (*Q*_Egge_*_r_*(8) = 20.82, *p* = 7.64×10^−03^; *Q*_IVW_(9) = 21.15, *p* = 1.20×10^−02^). The Weighted Mode and Weighted Median estimates were consistent with the results from IVW. The MR-Egger effect size was consistent with the IVW estimate (β_Egger_ = 0.444, 95% CI = −0.146 to 1.034, *p* = 6.03×10^−01^). The I^2^ for MR-Egger was 0.971 from NMD to loneliness. There was no evidence of directional horizontal pleiotropy according to the MR-Egger Intercept (Intercept = - 0.002, *p* = 7.31×10^−01^). Steiger filtering determined that all SNPs were operating in the correct direction. The forest plot in Fig. 4 shows the estimated causal effect for the 10 LD-independent SNPs used as instruments for MR analyses for NMD to loneliness. Additional MR analyses using 19 instruments and a p-value threshold of 5 × 10^−07^ (Supplementary Tables 5n – 5r, Figs. 4e– 4f) demonstrate consistency with our main MR analyses. Full MR results can be found in the Supplementary Material.

## 4 Discussion

The aetiology underlying the complex associations between loneliness, substance use and psychiatric disorders has remained elusive. In this work, we aimed to dissect their shared and non-shared genetic basis and explore directionality of effects, by combining multivariate genome-wide methods with Mendelian randomization. First, we found evidence of genetic overlap between loneliness and a latent genetic factor encompassing neurodevelopmental and mood conditions. Second, MR analyses results suggested a potentially bidirectional causal effect between loneliness and neurodevelopmental/mood conditions.

### 4.1 Unique genetic correlations between loneliness and neurodevelopmental and mood conditions

Exploring the genetic correlations between 11 traits indexing mental and behavioural health, we identified three latent genetic factors, including neurodevelopmental and mood conditions (NMD), substance use traits (SUT) and disorders with psychotic features (DPF). After controlling for the intercorrelations between the three latent factors, loneliness was found to be genetically most correlated with the latent factor encompassing neurodevelopmental and mood conditions. These findings are in line with other research using different methods which has demonstrated strong genetic associations between loneliness and mood disorders (Abdellaoui et al., 2019; Rødevand et al., 2021). Our findings are also in agreement with epidemiological and neurocognitive research suggesting that loneliness, neurodevelopmental and mood conditions share several common features, such as biased processing of interpersonal cues (Bar-Haim et al., 2007; Meng et al., 2020), poor emotional regulation (England-Mason, 2020; Eres et al., 2021) and internalizing symptoms like guilt, negative self-evaluation and social withdrawal (Bauminger et al., 2010; Jacob et al., 2014). In conclusion, this shared genetic aetiology between loneliness and neurodevelopmental and mood conditions might partly account for their co-occurrence and may also be indicative of a causal relationship between them (van Rheenen et al., 2019).

### 4.2 Causal effects between loneliness and neurodevelopmental and mood conditions

Addressing the second of our aims, we found evidence of a potentially causal effect from loneliness to neurodevelopmental and mood conditions based on consistent results across sensitivity analyses. Evidence of a causal effect in the reverse direction, from neurodevelopmental and mood conditions to loneliness, were also present, although there was evidence of heterogeneity.

The results of our MR analyses are in line with results from previous MR analyses. For example, Choi et al. (2020a) found that an increased ability to confide in others, a key feature of loneliness (Achterbergh et al., 2020; West et al., 1986), was causally related to decreased symptoms of depression. Our findings suggesting a downstream effect of loneliness on neurodevelopmental and mood conditions are also supported by existing meta-analyses examining the effects of loneliness on various mental health outcomes (Erzen and Çikrikci, 2018; Park et al., 2020).

Longitudinal studies demonstrating the adverse effects of loneliness on later measures of mental health outcomes also seem to corroborate our MR results. A recent systematic review preprint examined 20 longitudinal studies pertaining to the relationship between loneliness and the onset of mental health issues. Across these longitudinal studies, people who reported often feeling lonely at baseline were more likely to report onset of depression than those who did not often feel lonely (Mann et al., 2021 MedRxiv). Our findings also align with clinical Randomized Control Trials (RCTs) that have suggested the effectiveness of loneliness interventions in improving wellbeing and reducing depressive symptoms (Lai et al., 2020; Shapira et al., 2021). It is, however, unclear from these RCTs whether the amelioration of mood issues occurred as a direct consequence of interventions leading to reduced loneliness rather than to alterations in emotion regulation or cognition. A scoping review of literature addressing interventions targeting loneliness found that the most promising interventions were indeed those that targeted cognitive processing (Mann et al., 2017). It is possible, therefore, that interventions which target the maladaptive cognitions that contribute to loneliness subsequently relieve mood problems reported in neurodevelopmental and mood conditions due to downstream effects.

Our MR analyses also provide evidence of a potential causal effect of neurodevelopmental and mood conitions on loneliness, and as such, a bidirectional causal association. This bidirectional causal association may point toward a mutually reinforcing relationship between loneliness and neurodevelopmental and mood conditions. As suggested by a meta-synthesis of studies capturing experiences of loneliness amongst depressed individuals, neurodevelopmental and mood conditions may encourage behaviours such as social withdrawal that elevate feelings of loneliness. Subsequently, this may contribute to low mood, perpetuating a cycle of loneliness and mood problems (Achterbergh et al., 2020). However, our findings of a potential causal effect of neurodevelopmental and mood conditions on loneliness warrants replication as there was evidence of heterogeneity, which can inflate MR estimates.

### 4.3 Limitations and future considerations

Our findings highlight important considerations for future research. While the causal effect of loneliness on mood disorders suggested by our findings is well-supported by existing literature, the causal effect of loneliness on neurodevelopmental conditions is less well-established. For example, there is little to no research examining whether challenges unique to neurodevelopmental conditions worsen with increased loneliness. Loneliness may exacerbate existing communication difficulties amongst individuals with neurodevelopmental conditions, prevent the development of greater social competence (Koegel, 2000) and may also promote hyperactivity (Ouchi et al., 2013; Sullens et al., 2021). Despite this, existing literature with human subjects often only examines the effects of loneliness on symptoms of depression and anxiety amongst people with neurodevelopmental conditions. Regarding our analysis, the causal effect of loneliness on neurodevelopmental conditions may reflect the fact that mood problems are a key feature of neurodevelopmental conditions.

There are several limitations to consider when evaluating the results of this study. Firstly, the conceptualisation of latent factors can be subjective, and factor structures can often be unstable (Abdellaoui et al., 2021). This makes it challenging to compare findings across studies. While our factor structure closely resembles that of several genomic factor analytic studies (e.g., Lee et al., 2019, 2021; Wu et al., 2020; Abdellaoui et al., 2021), Grotzinger et al. (2019) proposed a different structure. We found depression and anxiety to cluster alongside ADHD, autism and PTSD; in their analysis, ADHD, autism, PTSD and problematic alcohol use clustered alongside one another in a latent factor separate from depression and anxiety. Accordingly, they suggested separate latent factors encompassing neurodevelopmental conditions and internalizing disorders, respectively. The discrepancy between our factor structure and theirs may be explained by our use of different GWAS summary statistics throughout our analyses. For instance, we excluded several phenotypes that were present in their study, such as anorexia, obsessive-compulsive disorder and Tourette's syndrome, because of our thresholds for statistical power. We also included several substance use traits that were not present in their analysis.

Another consideration is that loneliness and mood conditions, while clinically and qualitatively distinguishable, share common features and present in similar ways. For example, social withdrawal is a transdiagnostic feature of many mood and neurodevelopmental conditions, and is indeed a key feature of loneliness (Bellini et al., 2023; Mazurek, 2013). The resemblance in the presentation of loneliness and mood conditions is highlighted by the fact that assessment measures for mood conditions and those for loneliness often contain overlapping questionnaire items. For example, according to various diagnostic tools, experiences such as social withdrawal, low mood, negative feelings towards the self, and disruptions in social interactions are shared across PTSD, anxiety, autism, ADHD, and major depression (American Psychiatric Association, 2013; World Health Organization, 2019). Several questionnaire items on the UCLA Loneliness Scale also pertain to similar experiences (Russell et al., 1980). In this regard, loneliness and mood conditions may share features that are a challenge to delineate. Consequently, the strong degree of genetic correlation between loneliness and mood conditions we observed (β = 0.66) may partly reflect the fact that their respective GWAS capture genetic influences on overlapping experiences, such as social withdrawal Alternatively, our findings may reflect spurious bidirectional associations, which can arise in situations of high co-heritability (Darrous et al., 2021).

The current study has several strengths. Firstly, we tested a range of factor structures that were informed based both on data-driven approaches as well as on existing theory of how certain disorders tend to cluster together (Supplementary Note S3). A second strength related to our factor analysis procedure is that we avoided model over-fitting by conducting EFA and CFA on alternate chromosomes. Finally, a strength of our approach is that we considered the complex nature of associations between loneliness and psychiatric disorders and substance use by explicitly modelling their genetic intercorrelations in GSEM. This strength also extends to the bidirectional MR analysis: because we accounted for genetic overlap between the factors prior to conducting multivariable GWAS, we can be more certain that the causal effects from loneliness to neurodevelopmental and mood conditions do not reflect contributing effects from the other phenotypes we included.

## 4 Conclusion

We provide evidence of a genetic association between loneliness and a latent genetic factor representing five neurodevelopmental and mood conditions, over and above genetic overlap with other psychiatric disorder and substance use trait clusters. In addition, our findings suggest that loneliness may potentially exert downstream, causal effects on neurodevelopmental and mood conditions. Though we provide evidence supporting a causal effect of loneliness on neurodevelopmental and mood conditions, there was evidence of heterogeneity which may lead to inflated MR effects. We must addtionally consider that MR effects may have captured the overlap in the presentation of loneliness and symptoms of neurodevelopmental and mood conditions. Overall, genetic predisposition to loneliness may be an important factor associated with elevated risk of neurodevelopmental and mood conditions. In recent years, the prevalence of loneliness has been increasing substantially, especially considering social restrictions related to the Covid-19 pandemic (Greig et al., 2022; Li and Wang, 2020; Padmanabhanunni and Pretorius, 2021; Santini and Koyanagi, 2021). Given the increasing prevalence of loneliness and accumulating evidence of its causal effect on neurodevelopmental and mood problems, it is clear that loneliness should not be trivialised. Our findings highlight the importance of loneliness as an essential component of mental health prevention and policy.

## Author contribution statement

Ellen Martin: Conceptualization, Methodology, Visualization, Formal Analysis, Writing – Original Draft

Tabea Schoeler: Conceptualization, Methodology, Writing – Review and Editing

Wikus Barkhuizen: Conceptualization, Methodology, Supervision, Writing – Review and Editing

Jean-Baptiste Pingault: Conceptualization, Supervision, Writing – Review and Editing

## Declaration of Competing Interest

The authors declare that they have no known competing financial interests or personal relationships that could have appeared to influence the work reported in this paper.

## Appendix Supplementary materials

Image, application 1

## References

[bib0001] Abdellaoui A., Sanchez-Roige S., Sealock J., Treur J.L., Dennis J., Fontanillas P., Elson S., Nivard M.G., Ip H.F., van der Zee M., Baselmans B.M.L., Hottenga J.J., Willemsen G., Mosing M., Lu Y., Pedersen N.L., Denys D., Amin N., M Van Duijn C., Szilagyi I., Tiemeier H., Neumann A., Verweij K.J.H., Cacioppo S., Cacioppo J.T., Davis L.K., Palmer A.A., Boomsma D.I. (2019). Phenome-wide investigation of health outcomes associated with genetic predisposition to loneliness. Hum. Mol. Genet..

[bib0002] Abdellaoui A., Smit D.J.A., van den Brink W., Denys D., Verweij K.J.H. (2021). Genomic relationships across psychiatric disorders including substance use disorders. Drug Alcohol Depend..

[bib0003] Achterbergh L., Pitman A., Birken M., Pearce E., Sno H., Johnson S. (2020). The experience of loneliness among young people with depression: a qualitative meta-synthesis of the literature. BMC Psychiatry.

[bib0004] American Psychiatric Association (2013).

[bib0005] Bar-Haim Y., Lamy D., Pergamin L., Bakermans-Kranenburg M.J., van Ijzendoorn M.H. (2007). Threat-related attentional bias in anxious and nonanxious individuals: a meta-analytic study. Psychol. Bull..

[bib0006] Barreto M., Victor C., Hammond C., Eccles A., Richins M.T., Qualter P. (2021). Loneliness around the world: age, gender, and cultural differences in loneliness. Pers. Individ. Dif..

[bib0007] Bauminger N., Solomon M., Rogers S.J. (2010). Externalizing and internalizing behaviors in ASD. Autism Res..

[bib0008] Bellini B., Perrotti G., Gambolò L., Baglioni V., Faedda N., Natalucci G., Pezzuti L., Ardizzone I., Guidetti V. (2023). Prolonged social withdrawal during adolescence: transdiagnostic syndrome or a new psychiatric entity?. Child Psychiatry Hum. Dev..

[bib0009] Bowden J., Davey Smith G., Haycock P.C., Burgess S. (2016). Consistent estimation in mendelian randomization with some invalid instruments using a weighted median estimator. Genet. Epidemiol..

[bib0010] Bowden J., Smith G.D., Burgess S. (2015). Mendelian randomization with invalid instruments: effect estimation and bias detection through Egger regression. Int. J. Epidemiol..

[bib0011] Bulik-Sullivan B., Finucane H.K., Anttila V., Gusev A., Day F.R., Loh P.R., Duncan L., Perry J.R.B., Patterson N., Robinson E.B., Daly M.J., Price A.L., Neale B.M. (2015). An atlas of genetic correlations across human diseases and traits. Nat. Genet..

[bib0012] Burgess S., Bowden J., Fall T., Ingelsson E., Thompson S.G. (2017). Sensitivity analyses for robust causal inference from mendelian randomization analyses with multiple genetic variants. Epidemiology.

[bib0013] Burgess S., Butterworth A., Thompson S.G. (2013). Mendelian randomization analysis with multiple genetic variants using summarized data. Genet. Epidemiol..

[bib0014] Burgess S., Thompson S.G. (2017). Interpreting findings from Mendelian randomization using the MR-Egger method. Eur. J. Epidemiol..

[bib0015] Cacioppo J.T., Cacioppo S. (2018). The growing problem of loneliness. Lancet.

[bib0016] Cacioppo S., Grippo A.J., London S., Goossens L., Cacioppo J.T. (2015). Loneliness: clinical Import and Interventions. Perspect. Psychol. Sci..

[bib0017] Caspi A., Houts R.M., Belsky D.W., Goldman-Mellor S.J., Harrington H., Israel S., Meier M.H., Ramrakha S., Shalev I., Poulton R., Moffitt T.E. (2014). The p factor: one general psychopathology factor in the structure of psychiatric disorders?. Clin. Psychol. Sci..

[bib0018] Choi S.W., Mak T.S.H., O'Reilly P.F. (2020). Tutorial: a guide to performing polygenic risk score analyses. Nat. Protoc..

[bib0019] Copeland M., Fisher J.C., Moody J., Feinberg M.E. (2018). Different kinds of lonely: dimensions of isolation and substance use in adolescence. J. Youth Adolesc..

[bib0020] Darrous L., Mounier N., Kutalik Z. (2021). Simultaneous estimation of bi-directional causal effects and heritable confounding from GWAS summary statistics. Nat. Commun..

[bib0021] Day F.R., Ong K.K., Perry J.R.B. (2018). Elucidating the genetic basis of social interaction and isolation. Nat. Commun..

[bib0022] Demontis D., Walters R.K., Martin J., Mattheisen M., Als T.D., Agerbo E., Baldursson G., Belliveau R., Bybjerg-Grauholm J., Bækvad-Hansen M., Cerrato F., Chambert K., Churchhouse C., Dumont A., Eriksson N., Gandal M., Goldstein J.I., Grasby K.L., Grove J., Gudmundsson O.O., Hansen C.S., Hauberg M.E., Hollegaard M.V., Howrigan D.P., Huang H., Maller J.B., Martin A.R., Martin N.G., Moran J., Pallesen J., Palmer D.S., Pedersen C.B., Pedersen M.G., Poterba T., Poulsen J.B., Ripke S., Robinson E.B., Satterstrom F.K., Stefansson H., Stevens C., Turley P., Walters G.B., Won H., Wright M.J., Albayrak Ö., Anney R.J.L., Arranz M.J., Banaschewski T.J., Bau C., Biederman J., Buitelaar J.K., Casas M., Charach A., Crosbie J., Dempfle A., Doyle A.E., Ebstein R.P., Elia J., Freitag C., Föcker M., Gill M., Grevet E., Hawi Z., Hebebrand J., Herpertz-Dahlmann B., Hervas A., Hinney A., Hohmann S., Holmans P., Hutz M., Ickowitz A., Johansson S., Kent L., Kittel-Schneider S., Lambregts-Rommelse N., Lehmkuhl G., Loo S.K., McGough J.J., Meyer J., Mick E., Middletion F., Miranda A., Mota N.R., Mulas F., Mulligan A., Nelson F., Nguyen T.T., Oades R.D., O'Donovan M.C., Owen M.J., Palmason H., Ramos-Quiroga J.A., Renner T.J., Ribasés M., Rietschel M., Rivero O., Romanos J., Romanos M., Rothenberger A., Royers H., Sánchez-Mora C., Scherag A., Schimmelmann B.G., Schäfer H., Sergeant J., Sinzig J., Smalley S.L., Steinhausen H.C., Thompson M., Todorov A., Vasquez A.A., Walitza S., Wang Y., Warnke A., Williams N., Witt S.H., Yang L., Zayats T., Zhang-James Y., Smith G.D., Davies G.E., Ehli E.A., Evans D.M., Fedko I.O., Greven C.U., Groen-Blokhuis M.M., Guxens M., Hammerschlag A.R., Hartman C.A., Heinrich J., Jan Hottenga J., Hudziak J., Jugessur A., Kemp J.P., Krapohl E., Murcia M., Myhre R., Nolte I.M., Nyholt D.R., Ormel J., Ouwens K.G., Pappa I., Pennell C.E., Plomin R., Ring S., Standl M., Stergiakouli E., Pourcain B.S., Stoltenberg C., Sunyer J., Thiering E., Tiemeier H., Tiesler C.M.T., Timpson N.J., Trzaskowski M., van der Most P.J., Vilor-Tejedor N., Wang C.A., Whitehouse A.J.O., Zhao H., Agee M., Alipanahi B., Auton A., Bell R.K., Bryc K., Elson S.L., Fontanillas P., Furlotte N.A., Hinds D.A., Hromatka B.S., Huber K.E., Kleinman A., Litterman N.K., McIntyre M.H., Mountain J.L., Northover C.A.M., Pitts S.J., Sathirapongsasuti J.F., Sazonova O.V., Shelton J.F., Shringarpure S., Tian C., Vacic V., Wilson C.H., Andreassen O.A., Asherson P., Burton C.L., Boomsma D.I., Cormand B., Dalsgaard S., Franke B., Gelernter J., Geschwind D., Hakonarson H., Haavik J., Kranzler H.R., Kuntsi J., Langley K., Lesch K.P., Middeldorp C., Reif A., Rohde L.A., Roussos P., Schachar R., Sklar P., Sonuga-Barke E.J.S., Sullivan P.F., Thapar A., Tung J.Y., Waldman I.D., Medland S.E., Stefansson K., Nordentoft M., Hougaard D.M., Werge T., Mors O., Mortensen P.B., Daly M.J., Faraone S.V., Børglum A.D., Neale B.M. (2018). Discovery of the first genome-wide significant risk loci for attention deficit/hyperactivity disorder. Nat. Genet..

[bib0023] England-Mason G. (2020). Emotion regulation as a transdiagnostic feature in children with neurodevelopmental disorders. Curr. Dev. Disord. Rep..

[bib0024] Eres, R., Lim, M.H., Lanham, S., Jillard, C., Bates, G., 2021. Loneliness and emotion regulation: implications of having social anxiety disorder. 73, 46–56. 10.1080/00049530.2021.1904498.

[bib0025] Erzen E., Çikrikci Ö. (2018). The effect of loneliness on depression: a meta-analysis. Int. J. Soc. Psychiatry.

[bib0026] Fried L., Prohaska T., Burholt V., Burns A., Golden J., Hawkley L., Lawlor B., Leavey G., Lubben J., O'Sullivan R., Perissinotto C., van Tilburg T., Tully M., Victor C. (2020). A unified approach to loneliness. Lancet.

[bib0027] Gao J., Davis L.K., Hart A.B., Sanchez-Roige S., Han L., Cacioppo J.T., Palmer A.A. (2016). Genome-wide association study of loneliness demonstrates a role for common variation. Neuropsychopharmacology.

[bib0028] Giacco D. (2023). Loneliness and mood disorders: consequence, cause and/or unholy alliance?. Curr. Opin. Psychiatry.

[bib0029] Giacco D., Palumbo C., Strappelli N., Catapano F., Priebe S. (2016). Social contacts and loneliness in people with psychotic and mood disorders. Compr. Psychiatry.

[bib0030] Greig F., Perera G., Tsamakis K., Stewart R., Velayudhan L., Mueller C. (2022). Loneliness in older adult mental health services during the COVID-19 pandemic and before: associations with disability, functioning and pharmacotherapy. Int. J. Geriatr. Psychiatry.

[bib0031] Grotzinger A.D., Rhemtulla M., de Vlaming R., Ritchie S.J., Mallard T.T., Hill W.D., Ip H.F., Marioni R.E., McIntosh A.M., Deary I.J., Koellinger P.D., Harden K.P., Nivard M.G., Tucker-Drob E.M. (2019). Genomic structural equation modelling provides insights into the multivariate genetic architecture of complex traits. Nat. Hum. Behav..

[bib0032] Grove J., Ripke S., Als T.D., Mattheisen M., Walters R.K., Won H., Pallesen J., Agerbo E., Andreassen O.A., Anney R., Awashti S., Belliveau R., Bettella F., Buxbaum J.D., Bybjerg-Grauholm J., Bækvad-Hansen M., Cerrato F., Chambert K., Christensen J.H., Churchhouse C., Dellenvall K., Demontis D., De Rubeis S., Devlin B., Djurovic S., Dumont A.L., Goldstein J.I., Hansen C.S., Hauberg M.E., Hollegaard M.V., Hope S., Howrigan D.P., Huang H., Hultman C.M., Klei L., Maller J., Martin J., Martin A.R., Moran J.L., Nyegaard M., Nærland T., Palmer D.S., Palotie A., Pedersen C.B., Pedersen M.G., dPoterba T., Poulsen J.B., Pourcain B.S., Qvist P., Rehnström K., Reichenberg A., Reichert J., Robinson E.B., Roeder K., Roussos P., Saemundsen E., Sandin S., Satterstrom F.K., Davey Smith G., Stefansson H., Steinberg S., Stevens C.R., Sullivan P.F., Turley P., Walters G.B., Xu X., Wray N.R., Trzaskowski M., Byrne E.M., Abdellaoui A., Adams M.J., Air T.M., Andlauer T.F.M., Bacanu S.A., Beekman A.T.F., Bigdeli T.B., Binder E.B., Blackwood D.H.R., Bryois J., Buttenschøn H.N., Cai N., Castelao E., Clarke T.K., Coleman J.R.I., Colodro-Conde L., Couvy-Duchesne B., Craddock N., Crawford G.E., Davies G., Deary I.J., Degenhardt F., Derks E.M., Direk N., Dolan C.V., Dunn E.C., Eley T.C., Escott-Price V., Kiadeh F.F.H., Finucane H.K., Forstner A.J., Frank J., Gaspar H.A., Gill M., Goes F.S., Gordon S.D., Hall L.S., Hansen T.F., Herms S., Hickie I.B., Hoffmann P., Homuth G., Horn C., Hottenga J.J., Ising M., Jansen R., Jorgenson E., Knowles J.A., Kohane I.S., Kraft J., Kretzschmar W.W., Krogh J., Kutalik Z., Li Y., Lind P.A., MacIntyre D.J., MacKinnon D.F., Maier R.M., Maier W., Marchini J., Mbarek H., McGrath P., McGuffin P., Medland S.E., Mehta D., Middeldorp C.M., Mihailov E., Milaneschi Y., Milani L., Mondimore F.M., Montgomery G.W., Mostafavi S., Mullins N., Nauck M., Ng B., Nivard M.G., Nyholt D.R., O'Reilly P.F., Oskarsson H., Owen M.J., Painter J.N., Peterson R.E., Pettersson E., Peyrot W.J., Pistis G., Posthuma D., Quiroz J.A., Rice J.P., Riley B.P., Rivera M., Mirza S.S., Schoevers R., Schulte E.C., Shen L., Shi J., Shyn S.I., Sigurdsson E., Sinnamon G.C.B., Smit J.H., Smith D.J., Streit F., Strohmaier J., Tansey K.E., Teismann H., Teumer A., Thompson W., Thomson P.A., Thorgeirsson T.E., Traylor M., Treutlein J., Trubetskoy V., Uitterlinden A.G., Umbricht D., Van der Auwera S., van Hemert A.M., Viktorin A., Visscher P.M., Wang Y., Webb B.T., Weinsheimer S.M., Wellmann J., Willemsen G., Witt S.H., Wu Y., Xi H.S., Yang J., Zhang F., Arolt V., Baune B.T., Berger K., Boomsma D.I., Cichon S., Dannlowski U., de Geus E.J.C., DePaulo J.R., Domenici E., Domschke K., Esko T., Grabe H.J., Hamilton S.P., Hayward C., Heath A.C., Kendler K.S., Kloiber S., Lewis G., Li Q.S., Lucae S., Madden P.A.F., Magnusson P.K., Martin N.G., McIntosh A.M., Metspalu A., Müller-Myhsok B., Nöthen M.M., O'Donovan M.C., Paciga S.A., Pedersen N.L., Penninx B.W.J.H., Perlis R.H., Porteous D.J., Potash J.B., Preisig M., Rietschel M., Schaefer C., Schulze T.G., Smoller J.W., Tiemeier H., Uher R., Völzke H., Weissman M.M., Lewis C.M., Levinson D.F., Breen G., Agee M., Alipanahi B., Auton A., Bell R.K., Bryc K., Elson S.L., Fontanillas P., Furlotte N.A., Hromatka B.S., Huber K.E., Kleinman A., Litterman N.K., McIntyre M.H., Mountain J.L., Noblin E.S., Northover C.A.M., Pitts S.J., Sathirapongsasuti J.F., Sazonova O.V., Shelton J.F., Shringarpure S., Tung J.Y., Vacic V., Wilson C.H., Stefansson K., Geschwind D.H., Nordentoft M., Hougaard D.M., Werge T., Mors O., Mortensen P.B., Neale B.M., Daly M.J., Børglum A.D. (2019). Identification of common genetic risk variants for autism spectrum disorder. Nat. Genet..

[bib0033] Hartwig F.P., Smith G.D., Bowden J. (2017). Robust inference in summary data Mendelian randomization via the zero modal pleiotropy assumption. Int. J. Epidemiol..

[bib0034] Hawkley L.C., Cacioppo J.T. (2010). Loneliness matters: a theoretical and empirical review of consequences and mechanisms. Ann. Behav. Med..

[bib0035] Hemani G., Tilling K., Davey Smith G. (2017). Orienting the causal relationship between imprecisely measured traits using GWAS summary data. PLos Genet..

[bib0036] Houghton S., Lawrence D., Hunter S.C., Zadow C., Kyron M., Paterson R., Carroll A., Christie R., Brandtman M. (2020). Loneliness accounts for the association between diagnosed attention deficit-hyperactivity disorder and symptoms of depression among adolescents. J. Psychopathol. Behav. Assess..

[bib0037] Howard D.M., Adams M.J., Clarke T.K., Hafferty J.D., Gibson J., Shirali M., Coleman J.R.I., Hagenaars S.P., Ward J., Wigmore E.M., Alloza C., Shen X., Barbu M.C., Xu E.Y., Whalley H.C., Marioni R.E., Porteous D.J., Davies G., Deary I.J., Hemani G., Berger K., Teismann H., Rawal R., Arolt V., Baune B.T., Dannlowski U., Domschke K., Tian C., Hinds D.A., Trzaskowski M., Byrne E.M., Ripke S., Smith D.J., Sullivan P.F., Wray N.R., Breen G., Lewis C.M., McIntosh A.M. (2019). Genome-wide meta-analysis of depression identifies 102 independent variants and highlights the importance of the prefrontal brain regions. Nat. Neurosci..

[bib0038] Ingram I., Kelly P.J., Deane F.P., Baker A.L., Goh M.C.W., Raftery D.K., Dingle G.A. (2020). Loneliness among people with substance use problems: a narrative systematic review. Drug Alcohol Rev..

[bib0039] Ingram I., Kelly P.J., Haslam C., O'Neil O.J., Deane F.P., Baker A.L., Dingle G.A. (2020). Reducing loneliness among people with substance use disorders: feasibility of ‘Groups for Belonging. Drug Alcohol Rev..

[bib0040] Jacob C., Gross-Lesch S., Jans T., Geissler J., Reif A., Dempfle A., Lesch K.P. (2014). Internalizing and externalizing behavior in adult ADHD. ADHD Attention Deficit Hyperactivity Disord..

[bib0041] Jang S.K., Saunders G., Liu M.Z., Jiang Y., Liu D.J., Vrieze S. (2022). Genetic correlation, pleiotropy, and causal associations between substance use and psychiatric disorder. Psychol. Med..

[bib0042] Johnson E.C., Demontis D., Thorgeirsson T.E., Walters R.K., Polimanti R., Hatoum A.S., Sanchez-Roige S., Paul S.E. (2020). A large-scale genome-wide association study meta-analysis of cannabis use disorder. Lancet Psychiatry.

[bib0043] Koegel L.K. (2000). Interventions to facilitate communication in autism. J. Autism. Dev. Disord..

[bib0044] Lai D.W.L., Li J., Ou X., Li C.Y.P. (2020). Effectiveness of a peer-based intervention on loneliness and social isolation of older Chinese immigrants in Canada: a randomized controlled trial. BMC Geriatr..

[bib0045] Leathem L.D., Currin D.L., Montoya A.K., Karlsgodt K.H. (2021). Socioemotional mechanisms of loneliness in subclinical psychosis. Schizophr. Res..

[bib0046] Lee P.H., Anttila V., Won H., Feng Y.C.A., Rosenthal J., Zhu Z., Tucker-Drob E.M., Nivard M.G. (2019). Genomic relationships, novel loci, and pleiotropic mechanisms across eight psychiatric disorders. CellCell.

[bib0047] Lee P.H., Feng Y.C.A., Smoller J.W. (2021). Pleiotropy and cross-disorder genetics among psychiatric disorders. Biol. Psychiatry.

[bib0048] Leigh-Hunt N., Bagguley D., Bash K., Turner V., Turnbull S., Valtorta N., Caan W. (2017). An overview of systematic reviews on the public health consequences of social isolation and loneliness. Public Health.

[bib0049] Li L.Z., Wang S. (2020). Prevalence and predictors of general psychiatric disorders and loneliness during COVID-19 in the United Kingdom. Psychiatry Res..

[bib0050] Lim M.H., Gleeson J.F.M., Alvarez-Jimenez M., Penn D.L. (2018). Loneliness in psychosis: a systematic review. Soc. Psychiatry Psychiatr. Epidemiol..

[bib0051] Mann F., Bone J.K., Lloyd-Evans B., Frerichs J., Pinfold V., Ma R., Wang J., Johnson S. (2017). A life less lonely: the state of the art in interventions to reduce loneliness in people with mental health problems. Soc. Psychiatry Psychiatr. Epidemiol..

[bib0052] Mann F., Wang J., Pearce E., Ma R., Schleif M., Lloyd-Evans B., Johnson S. (2021). Loneliness and the onset of new mental health problems in the general population: a systematic review. medRxiv.

[bib0053] Matthews T., Danese A., Caspi A., Fisher H.L., Goldman-Mellor S., Kepa A., Moffitt T.E., Odgers C.L., Arseneault L. (2019). Lonely young adults in modern Britain: findings from an epidemiological cohort study. Psychol. Med..

[bib0054] Mazurek, M.O., 2013. Loneliness, friendship, and well-being in adults with autism spectrum disorders. 18, 223–232. 10.1177/1362361312474121. 24092838

[bib0055] Meng J., Wang X., Wei D., Qiu J. (2020). State loneliness is associated with emotional hypervigilance in daily life: a network analysis. Pers. Individ. Dif..

[bib0056] Nievergelt C.M., Maihofer A.X., Klengel T., Atkinson E.G., Chen C.Y., Choi K.W., Coleman J.R.I., Dalvie S., Duncan L.E., Gelernter J., Levey D.F., Logue M.W., Polimanti R., Provost A.C., Ratanatharathorn A., Stein M.B., Torres K., Aiello A.E., Almli L.M., Amstadter A.B., Andersen S.B., Andreassen O.A., Arbisi P.A., Ashley-Koch A.E., Austin S.B., Avdibegovic E., Babić D., Bækvad-Hansen M., Baker D.G., Beckham J.C., Bierut L.J., Bisson J.I., Boks M.P., Bolger E.A., Børglum A.D., Bradley B., Brashear M., Breen G., Bryant R.A., Bustamante A.C., Bybjerg-Grauholm J., Calabrese J.R., Caldas- de- Almeida J.M., Sheerin C.M., Silove D., Smith A.K., Smoller J.W., Sponheim S.R., Stein D.J., Stevens J.S., Sumner J.A., Teicher M.H., Thompson W.K., Trapido E., Uddin M., Ursano R.J., van den Heuvel L.L., Van Hooff M., Vermetten E., Vinkers C.H., Voisey J., Wang Y., Wang Z., Werge T., Williams M.A., Williamson D.E., Winternitz S., Wolf C., Wolf E.J., Wolff J.D., Yehuda R., Young R.M.D., Young K.A., Zhao H., Zoellner L.A., Liberzon I., Ressler K.J., Haas M., Koenen K.C. (2019). International meta-analysis of PTSD genome-wide association studies identifies sex- and ancestry-specific genetic risk loci. Nat. Commun..

[bib0057] Otowa T., Hek K., Lee M., Byrne E.M., Mirza S.S., Nivard M.G., Bigdeli T., Aggen S.H., Adkins D., Wolen A., Fanous A., Keller M.C., Castelao E., Kutalik Z., Der Auwera S.V., Homuth G., Nauck M., Teumer A., Milaneschi Y., Hottenga J.J., Direk N., Hofman A., Uitterlinden A., Mulder C.L., Henders A.K., Medland S.E., Gordon S., Heath A.C., Madden P.A.F., Pergadia M.L., Van Der Most P.J., Nolte I.M., Van Oort F.V.A., Hartman C.A., Oldehinkel A.J., Preisig M., Grabe H.J., Middeldorp C.M., Penninx B.W.J.H., Boomsma D., Martin N.G., Montgomery G., Maher B.S., Van Den Oord E.J., Wray N.R., Tiemeier H., Hettema J.M. (2016). Meta-analysis of genome-wide association studies of anxiety disorders. Mol. Psychiatry.

[bib0058] Ouchi H., Ono K., Murakami Y., Matsumoto K. (2013). Social isolation induces deficit of latent learning performance in mice: a putative animal model of attention deficit/hyperactivity disorder. Behav. Brain Res..

[bib0059] Padmanabhanunni A., Pretorius T.B. (2021). The unbearable loneliness of COVID-19: cOVID-19-related correlates of loneliness in South Africa in young adults. Psychiatry Res..

[bib0060] Pardiñas A.F., Holmans P., Pocklington A.J., Escott-Price V., Ripke S., Carrera N., Legge S.E., Bishop S., Cameron D., Hamshere M.L., Han J., Hubbard L., Lynham A., Mantripragada K., Rees E., MacCabe J.H., McCarroll S.A., Baune B.T., Breen G., Byrne E.M., Dannlowski U., Eley T.C., Hayward C., Martin N.G., McIntosh A.M., Plomin R., Porteous D.J., Wray N.R., Caballero A., Geschwind D.H., Huckins L.M., Ruderfer D.M., Santiago E., Sklar P., Stahl E.A., Won H., Agerbo E., Als T.D., Andreassen O.A., Bækvad-Hansen M., Mortensen P.B., Pedersen Carsten Bøcker, Børglum A.D., Bybjerg-Grauholm J., Djurovic S., Durmishi N., Pedersen M.G., Golimbet V., Grove J., Hougaard D.M., Mattheisen M., Molden E., Mors O., Nordentoft M., Pejovic-Milovancevic M., Sigurdsson E., Silagadze T., Hansen C.S., Stefansson K., Stefansson H., Steinberg S., Tosato S., Werge T., Harold D., Sims R., Gerrish A., Chapman J., Abraham R., Hollingworth P., Pahwa J., Denning N., Thomas C., Taylor S., Powell J., Proitsi P., Lupton M., Lovestone S., Passmore P., Craig D., McGuinness B., Johnston J., Todd S., Maier W., Jessen F., Heun R., Schurmann B., Ramirez A., Becker T., Herold C., Lacour A., Drichel D., Nothen M., Goate A., Cruchaga C., Nowotny P., Morris J.C., Mayo K., O'Donovan M.C., Owen M.J., Williams J., Achilla E., Barr C.L., Böttger T.W., Cohen D., Curran S., Dempster E., Dima D., Sabes-Figuera R., Flanagan R.J., Frangou S., Frank J., Gasse C., Gaughran F., Giegling I., Hannon E., Hartmann A.M., Heißerer B., Helthuis M., Horsdal H.T., Ingimarsson O., Jollie K., Kennedy J.L., Köhler O., Konte B., Lang M., Lewis C., MacCaba J., Malhotra A.K., McCrone P., Meier S.M., Mill J., Nöthen M.M., Pedersen C.B., Rietschel M., Rujescu D., Schwalber A., Sørensen H.J., Spencer B., Støvring H., Strohmaier J., Sullivan P., Vassos E., Verbelen M., Collier D.A., Kirov G., Walters J.T.R. (2018). Common schizophrenia alleles are enriched in mutation-intolerant genes and in regions under strong background selection. Nat. Genet..

[bib0061] Park, C., Majeed, A., Gill, H., Lee, Y., Tamura, J., Ho, R.C., Mansur, R.B., Nasri, F., Rosenblat, J.D., Wong, E., Mcintyre, R.S., 2020. The Effect of Loneliness on Distinct Health Outcomes: a Comprehensive Review and Meta-Analysis. 10.2139/ssrn.3463317. 33130511

[bib0062] R Core Team, 2022. R: a Language and Environment for Statistical Computing.

[bib0063] Richmond R.C., Smith G.D. (2022). Mendelian randomization: concepts and scope. Cold Spring Harb. Perspect. Med..

[bib0064] Rødevand L., Bahrami S., Frei O., Lin A., Gani O., Shadrin A., Smeland O.B., Connell K.S.O., Elvsåshagen T., Winterton A., Quintana D.S., Hindley G.F.L., Werner M.C.F., Djurovic S., Dale A.M., Lagerberg T.V., Steen N.E., Andreassen O.A. (2021). Polygenic overlap and shared genetic loci between loneliness, severe mental disorders, and cardiovascular disease risk factors suggest shared molecular mechanisms. Transl. Psychiatry.

[bib0065] Rodney T., Josiah N., Baptiste D.L. (2021). Loneliness in the time of COVID-19: impact on older adults. J. Adv. Nurs..

[bib0066] Rosseel Y. (2012). lavaan: an R package for structural equation modeling. J. Stat. Softw..

[bib0067] Russell D., Peplau L.A., Cutrona C.E. (1980). The revised UCLA loneliness scale: concurrent and discriminant validity evidence. J. Pers. Soc. Psychol..

[bib0068] Santini Z.I., Koyanagi A. (2021). Loneliness and its association with depressed mood, anxiety symptoms, and sleep problems in Europe during the COVID-19 pandemic. Acta Neuropsychiatr..

[bib0069] Schumann G., Liu C., O'Reilly P., Gao H., Song P., Xu B., Ruggeri B., Amin N., Jia T., Preis S., Lepe M.S., Akira S., Barbieri C., Baumeister S., Cauchi S., Clarke T.K., Enroth S., Fischer K., Hällfors J., Harris S.E., Hieber S., Hofer E., Hottenga J.J., Johansson Å., Joshi P.K., Kaartinen N., Laitinen J., Lemaitre R., Loukola A., Luan J., Lyytikäinen L.P., Mangino M., Manichaikul A., Mbarek H., Milaneschi Y., Moayyeri A., Mukamal K., Nelson C., Nettleton J., Partinen E., Rawal R., Robino A., Rose L., Sala C., Satoh T., Schmidt R., Schraut K., Scott R., Smith A.V., Starr J.M., Teumer A., Trompet S., Uitterlinden A.G., Venturini C., Vergnaud A.C., Verweij N., Vitart V., Vuckovic D., Wedenoja J., Yengo L., Yu B., Zhang W., Zhao J.H., Boomsma D.I., Chambers J., Chasman D.I., Daniela T., De Geus E., Deary I., Eriksson J.G., Esko T., Eulenburg V., Franco O.H., Froguel P., Gieger C., Grabe H.J., Gudnason V., Gyllensten U., Harris T.B., Hartikainen A.L., Heath A.C., Hocking L., Hofman A., Huth C., Jarvelin M.R., Jukema J.W., Kaprio J., Kooner J.S., Kutalik Z., Lahti J., Langenberg C., Lehtimäki T., Liu Y., Madden P.A.F., Martin N., Morrison A., Penninx B., Pirastu N., Psaty B., Raitakari O., Ridker P., Rose R., Rotter J.I., Samani N.J., Schmidt H., Spector T.D., Stott D., Strachan D., Tzoulaki I., Van Der Harst P., Van Duijn C.M., Marques-Vidal P., Vollenweider P., Wareham N.J., Whitfield J.B., Wilson J., Wolffenbuttel B., Bakalkin G., Evangelou E., Liu Yun, Rice K.M., Desrivières S., Kliewer S.A., Mangelsdorf D.J., Müller C.P., Levy D., Elliott P. (2016). KLB is associated with alcohol drinking, and its gene product β-Klotho is necessary for FGF21 regulation of alcohol preference. Proc. Natl. Acad. Sci. U. S. A..

[bib0070] Shapira S., Yeshua-Katz D., Cohn-Schwartz E., Aharonson-Daniel L., Sarid O., Clarfield A.M. (2021). A pilot randomized controlled trial of a group intervention via Zoom to relieve loneliness and depressive symptoms among older persons during the COVID-19 outbreak. Internet Interv..

[bib0071] Stahl E.A., Breen G., Forstner A.J., McQuillin A., Ripke S., Trubetskoy V., Mattheisen M., Wang Y., Coleman J.R.I., Gaspar H.A., de Leeuw C.A., Steinberg S., Pavlides J.M.W., Trzaskowski M., Byrne E.M., Pers T.H., Holmans P.A., Richards A.L., Abbott L., Agerbo E., Akil H., Albani D., Alliey-Rodriguez N., Als T.D., Anjorin A., Antilla V., Awasthi S., Badner J.A., Bækvad-Hansen M., Barchas J.D., Bass N., Bauer M., Belliveau R., Bergen S.E., Pedersen C.B., Bøen E., Boks M.P., Boocock J., Budde M., Bunney W., Burmeister M., Bybjerg-Grauholm J., Byerley W., Casas M., Cerrato F., Cervantes P., Chambert K., Charney A.W., Chen D., Churchhouse C., Clarke T.K., Coryell W., Craig D.W., Cruceanu C., Curtis D., Czerski P.M., Dale A.M., de Jong S., Degenhardt F., Del-Favero J., DePaulo J.R., Djurovic S., Dobbyn A.L., Dumont A., Elvsåshagen T., Escott-Price V., Fan C.C., Fischer S.B., Flickinger M., Foroud T.M., Forty L., Frank J., Fraser C., Freimer N.B., Frisén L., Gade K., Gage D., Garnham J., Giambartolomei C., Pedersen M.G., Goldstein J., Gordon S.D., Gordon-Smith K., Green E.K., Green M.J., Greenwood T.A., Grove J., Guan W., Guzman-Parra J., Hamshere M.L., Hautzinger M., Heilbronner U., Herms S., Hipolito M., Hoffmann P., Holland D., Huckins L., Jamain S., Johnson J.S., Juréus A., Kandaswamy R., Karlsson R., Kennedy J.L., Kittel-Schneider S., Knowles J.A., Kogevinas M., Koller A.C., Kupka R., Lavebratt C., Lawrence J., Lawson W.B., Leber M., Lee P.H., Levy S.E., Li J.Z., Liu C., Lucae S., Maaser A., MacIntyre D.J., Mahon P.B., Maier W., Martinsson L., McCarroll S., McGuffin P., McInnis M.G., McKay J.D., Medeiros H., Medland S.E., Meng F., Milani L., Montgomery G.W., Morris D.W., Mühleisen T.W., Mullins N., Nguyen H., Nievergelt C.M., Adolfsson A.N., Nwulia E.A., O'Donovan C., Loohuis L.M.O., Ori A.P.S., Oruc L., Ösby U., Perlis R.H., Perry A., Pfennig A., Potash J.B., Purcell S.M., Regeer E.J., Reif A., Reinbold C.S., Rice J.P., Rivas F., Rivera M., Roussos P., Ruderfer D.M., Ryu E., Sánchez-Mora C., Schatzberg A.F., Scheftner W.A., Schork N.J., Shannon Weickert C., Shehktman T., Shilling P.D., Sigurdsson E., Slaney C., Smeland O.B., Sobell J.L., Søholm Hansen C., Spijker A.T., St Clair D., Steffens M., Strauss J.S., Streit F., Strohmaier J., Szelinger S., Thompson R.C., Thorgeirsson T.E., Treutlein J., Vedder H., Wang W., Watson S.J., Weickert T.W., Witt S.H., Xi S., Xu W., Young A.H., Zandi P., Zhang P., Zöllner S., Adolfsson R., Agartz I., Alda M., Backlund L., Baune B.T., Bellivier F., Berrettini W.H., Biernacka J.M., Blackwood D.H.R., Boehnke M., Børglum A.D., Corvin A., Craddock N., Daly M.J., Dannlowski U., Esko T., Etain B., Frye M., Fullerton J.M., Gershon E.S., Gill M., Goes F., Grigoroiu-Serbanescu M., Hauser J., Hougaard D.M., Hultman C.M., Jones I., Jones L.A., Kahn R.S., Kirov G., Landén M., Leboyer M., Lewis C.M., Li Q.S., Lissowska J., Martin N.G., Mayoral F., McElroy S.L., McIntosh A.M., McMahon F.J., Melle I., Metspalu A., Mitchell P.B., Morken G., Mors O., Mortensen P.B., Müller-Myhsok B., Myers R.M., Neale B.M., Nimgaonkar V., Nordentoft M., Nöthen M.M., O'Donovan M.C., Oedegaard K.J., Owen M.J., Paciga S.A., Pato C., Pato M.T., Posthuma D., Ramos-Quiroga J.A., Ribasés M., Rietschel M., Rouleau G.A., Schalling M., Schofield P.R., Schulze T.G., Serretti A., Smoller J.W., Stefansson H., Stefansson K., Stordal E., Sullivan P.F., Turecki G., Vaaler A.E., Vieta E., Vincent J.B., Werge T., Nurnberger J.I., Wray N.R., Di Florio A., Edenberg H.J., Cichon S., Ophoff R.A., Scott L.J., Andreassen O.A., Kelsoe J., Sklar P. (2019). Genome-wide association study identifies 30 loci associated with bipolar disorder. Nat. Genet..

[bib0072] Sullens D.G., Gilley K., Jensen K., Vichaya E., Dolan S.L., Sekeres M.J. (2021). Social isolation induces hyperactivity and exploration in aged female mice. PLoS ONE.

[bib0073] Surkalim D.L., Luo M., Eres R., Gebel K., van Buskirk J., Bauman A., Ding D. (2022). The prevalence of loneliness across 113 countries: systematic review and meta-analysis. BMJ.

[bib0074] Tu Y.K., Gunnell D., Gilthorpe M.S. (2008). Simpson's paradox, lord's paradox, and suppression effects are the same phenomenon - the reversal paradox. Emerg. Themes. Epidemiol..

[bib0075] van Rheenen W., Peyrot W.J., Schork A.J., Lee S.H., Wray N.R. (2019). Genetic correlations of polygenic disease traits: from theory to practice. Nat. Rev. Genet..

[bib0076] Vogel D.L., Bitman R.L., Hammer J.H., Wade N.G. (2013). Is stigma internalized? The longitudinal impact of public stigma on self-stigma. J. Couns. Psychol..

[bib0077] Wang J., Lloyd-Evans B., Marston L., Mann F., Ma R., Johnson S. (2020). Loneliness as a predictor of outcomes in mental disorders among people who have experienced a mental health crisis: a 4-month prospective study. BMC Psychiatry.

[bib0078] Wang J., Mann F., Lloyd-Evans B., Ma R., Johnson S. (2018). Associations between loneliness and perceived social support and outcomes of mental health problems: a systematic review. BMC Psychiatry.

[bib0079] West D.A., Kellner R., Moore-West M. (1986). The effects of loneliness: a review of the literature. Compr. Psychiatry.

[bib0080] Wootton R.E., Richmond R.C., Stuijfzand B.G., Lawn R.B., Sallis H.M., Taylor G.M.J., Hemani G., Jones H.J., Zammit S., Davey Smith G., Munafò M.R. (2020). Evidence for causal effects of lifetime smoking on risk for depression and schizophrenia: a Mendelian randomisation study. Psychol. Med..

[bib0081] World Health Organization, 2019. International statistical classification of diseases and related health problems, 11th ed. 10.2471/BLT.25.294650 PMC13531092 42683092

[bib0082] Wu Y., Cao H., Baranova A., Huang H., Li S., Cai L., Rao S., Dai M., Xie M., Dou Y., Hao Q., Zhu L., Zhang X., Yao Y., Xu M., Wang Q., Zhang F. (2020). Multi-trait analysis for genome-wide association study of five psychiatric disorders. Transl. Psychiatry.

[bib0083] Zhao Q., Wang J., Hemani G., Bowden J., Small D.S. (2018). Two sample Mendelian randomization using robust adjusted profile score [R package mr.raps version 0.2]. Ann. Stat..

