# Supplementary material for: Understanding the relationship between loneliness, substance use traits and psychiatric disorders: A genetically informed approach
Source: Psychiatry Res. 2023 Jul;325:115218. doi: 10.1016/j.psychres.2023.115218 (PMC10636586; doi:10.1016/j.psychres.2023.115218)
Supplement: Supplementary file 1 [file mmc1.docx]

**Supplementary Materials**

**Understanding the relationship between loneliness, substance use traits and psychiatric disorders: a genetically informed approach**

MARTIN, E., SCHOELER, T., PINGAULT, J-B., & BARKHUIZEN, W.

**Contents**

**S1 GWAS Summary Statistics1**

**S2 Quality Control Procedures3**

**S3 Factor Analysis Information4**

Description of Factor Analysis Procedure**4**

Factor Analysis Supplemental Results**5**

**S4 Genomic Structural Equation Model Specifications11**

**S5 Multivariate Genome-Wide Association Study15**

Description F1/NMD Multivariate GWAS**15**

Multivariate GWAS Summary Statistics**16**

**S6 Bidirectional Mendelian Randomization17**

Description MR Methodology**17**

MR Loneliness to NMD**18**

MR NMD (Q_SNP_ filtered) to Loneliness**22**

MR Loneliness (p < 5x10^-07^) to NMD…………………………………………………………………………………….**25**

MR NMD (Q_SNP_ filtered, p < 5x10^-07^) to Loneliness**28**

**S7 Discussion Supplement31**

**References32**

**S1 GWAS SUMMARY STATISTICS**

Supplementary Table 1. Table of GWAS summary statistics and F1 neurodevelopmental/mood latent genomic factor.

| **Phenotype** | **N** | **Source** | **SE** | ***h*2** | **Sample Prev.** | **Population Prev.** | **Measure** | **Power** | **Cases** | **Controls** | **MAF/INFO** | **Effect** |
| --- | --- | --- | --- | --- | --- | --- | --- | --- | --- | --- | --- | --- |
| ADHD | 55374 | Demontis et al (2018) | 0.015 | 0.236 | 0.364 | 0.050 | binary | 15.6 | 19099 | 34194 | no MAF | BETA |
| Alcohol Consumption | 70460 | Schumann (2015) | 0.008 | 0.050 | NA | NA | continuous | 6.3 | NA | NA | no INFO | BETA |
| Anxiety | 15730 | Otowa et al (2016) | 0.030 | 0.079 | 0.405 | 0.209 | binary | 2.6 | 7016 | 14745 | no MAF | BETA |
| Autism | 46350 | Grove et al (2019) | 0.010 | 0.118 | 0.397 | 0.012 | binary | 11.8 | 18381 | 27969 | no MAF | OR |
| Bipolar Disorder | 51710 | Stahl et al (2019) | 0.008 | 0.170 | 0.394 | 0.020 | binary | 22.6 | 20352 | 31358 | both present | OR |
| Cannabis Use Disorder | 364701 | Johnson et al (2020) | 0.006 | 0.070 | 0.054 | 0.010 | binary | 11.7 | 20916 | 363116 | no INFO, no MAF | OR |
| Loneliness | 445024 | Day et al (2018) | 0.002 | 0.042 | NA | NA | continuous | 21.9 | NA | NA | both present | BETA |
| Major Depressive Disorder | 807553 | Howard et al (2019) | 0.003 | 0.089 | 0.305 | 0.206 | binary | 29.7 | 246363 | 561190 | both present | BETA |
| PTSD | 9537 | Nievergelt et al (2019) | NA | 0.050 | 0.133 | 0.068 | binary | NA | 2424 | 7113 | both present | OR |
| Smoking | 462690 | Wooton et al (2019) | 0.003 | 0.090 | NA | NA | continuous | 29.5 | NA | NA | both present | BETA |
| Schizophrenia | 105318 | Pardiñas et al (2018) | 0.014 | 0.412 | 0.386 | 0.010 | binary | 30.3 | 40675 | 64643 | both present | OR |
| Neurodevelopmental and Mood Disorders Latent Factor (F1/NMD) | 378372 | Present Study |  |  | NA | NA | continuous | | NA | NA |  | BETA |

*Note.* N = Sample Size; SE = Standard Error; *h*2 = heritability index; MAF = Minor Allele Frequency; INFO = Imputation Quality; NMD = Neurodevelopmental and Mood Disorders latent factor.

**Description of GWAS summary statistics measurements**

ADHD (Demontis et al., 2018) was measured using the 10^th^ Edition of the International Classification of Diseases (ICD-10). Alcohol (Schumann et al., 2016) was measured as the amount of alcohol consumed in grams. Anxiety summary statistics (Otowa et al., 2016) came from a meta-analysis of six previous cohorts and measured based on any lifetime clinician diagnosis of an anxiety disorder. Autism (Grove et al., 2019) was based on psychiatrist diagnoses using the ICD-10 and included diagnoses of childhood autism, atypical autism, Asperger’s syndrome, other pervasive developmental disorders and unspecified pervasive developmental disorder. Bipolar disorder (Stahl et al., 2019) summary statistics came from 32 different cohorts in which cases were included if they had a lifetime diagnosis of bipolar disorder according to ICD-10 or DSM-IV. Cannabis use disorder (Johnson et al., 2020) was measured based on ICD-10 and DSM-IV for either lifetime cannabis use or cannabis dependence, and was clinician diagnosed. Loneliness (Day, Ong, & Perry, 2018) was measured continuously using the UCLA Loneliness scale. Major depressive disorder summary statistics came from a genome-wide meta-analysis including data from several previous studies. Depression (Howard et al., 2019) was measured based on a combination of measures including using a “broad definition of depression”, lifetime clinician diagnosis of depression and a history of receiving treatment for depression. PTSD (Nievergelt et al., 2019) summary statistics came from a genome-wide meta-analysis of several studies and was measured based on a clinician diagnosis of lifetime or current PTSD, according to DSM-III, DSM-IV and DSM-5. Smoking (Wootton et al., 2020) was measured continuously using a combined index representing individuals’ current smoking status, their frequency of smoking, smoking duration and smoking cessation. Finally, schizophrenia (Pardiñas et al., 2018) was measured based on DSM-IV clinically diagnosed schizophrenia or schizoaffective disorder.

**S2 QUALITY CONTROL PROCEDURES**

**Description of summary statistics selection and pre-processing**

We selected GWAS based on the largest possible sample size, on heritability (SNP-*h*2 > 0.05) and on heritability *z*-scores (SNP-*h2-z* > 2).

Quality control procedures following GWAS selection included subsetting data and renaming the columns so that they would be in the correct format for the GenomicSEM *munge()* function.

GSEM requires, at minimum, data pertaining to the positions of the SNPs on the chromosomes, the effect alleles (which are the alleles studied in relation to the trait), the alternative alleles, *beta* or odds ratio (*OR*) variant effect sizes (depending on whether the trait was measured continuously or categorically, respectively), *p*-values and the SNP IDs. The sample sizes were checked to ensure that they were consistent with the *N*’s reported by the original authors of the respective GWASs.

All OR effects were converted to *beta* effects using log(OR). This was to facilitate later analyses which involved looping functions over the 11 phenotypes.

***Munging***

The *munge()* function from the GenomicSEM package was used to compare the SNPs in the summary data with those included in the reference panel, *HapMap3* from <https://www.sanger.ac.uk/data/hapmap-3/> (Altshuler et al., 2010). This standardises the SNPs across the 11 phenotypes and excludes data with a minor allele frequency (MAF) less than 1% provided MAF information was available. Data were also filtered based on their imputation quality (INFO) where available, including any SNPs with INFO > 90%, to exclude genetic variants less reliably genotyped.

**S3 SUPPMENTARY FACTOR ANALYSIS INFORMATION**

First the method of factor analysis is described, then the full results provided.

**Description of factor analysis procedure**

***Splitting of LDSC output into ODD and EVEN chromosomes***

GSEM uses linkage disequilibrium score regression (LDSC) (Bulik-Sullivan et al., 2015) to estimate the genetic covariation across the 11 phenotypes. For factor analysis, loneliness was not included in the covariance matrix. The LDSC covariance matrix was split into two data sets, one including data of only odd chromosomes, the other including that of only even chromosomes

Exploratory Factor Analysis was executed using the odd chromosomes, with Confirmatory Factor Analysis conducted on even chromosomes. This was to prevent model over-fitting. A cut-off of .3 was imposed to aid the construction of a more simple, interpretable model where only traits with loadings greater than .3 were included in the CFA model specification.

Using the LDSC output, we generated a correlation heatmap to visually indicate the extent of genetic correlation using the *corrplot()* function from the *corrplot* package (version .84) (Taiyun Wei et al., 2017).

***Principal Component Analysis***

To first identify the number of latent genetic structures which might underlie the included traits, Principal Component Analysis (PCA) was carried out using the odd LDSC variance-covariance matrix using the *prcomp()* and *fviz_eig()* functions from the *factoextra* package, version 1.0.7 (Kassambara & Mundt, 2020). The number of factors to retain was determined by referring to Kaiser’s Criterion and Cattell’s scree plot for guidance.

***Exploratory Factor Analysis***

To establish the correspondence between the components and the traits, Exploratory Factor Analysis (EFA) was executed using the *fa()* function from the *psych* package (Revelle, 2020), retaining the number of factors recommended by PCA. A *Promax* rotation was specified which produces simpler, more interpretable factor structures than varimax when data are correlated (Finch, 2006). Additionally, the factor method specified was *Unweighted Least Squares* which provides more accurate factor loadings than both *Diagonally Weighted Least Squares* or *Maximum Likelihood* estimators (Forero et al., 2009).

The *fa()* function also printed the factor loadings which were used to inform the structure of the latent factor model. A guiding threshold of .3 was imposed to aid the construction of a more simple, interpretable model where traits with loadings greater than .3 were included in CFA. This was not a strict criterion, particularly if, based on strong theoretical grounds it made more sense for certain traits to load alongside others (e.g., smoking alongside alcohol and cannabis use on page 7).

***Confirmatory Factor analysis***

CFA was carried out with the GSEM *usermodel()* function, using the complete LDSC output*.* The *Diagonally Weighted Least Squares* estimator was used over *Maximum Likelihood* to estimate correlations between the latent variables due to its superior accuracy (Li, 2016). The fit indices chosen were the Standardised Root Mean Residual (SRMR) and the Comparative Fit Index (CFI). SRMR values less than or equal to .08 are deemed good fits (Hu & Bentler, 2009)with values below .10 being reasonable (Abdellaoui, Smit, van den Brink, Denys, & Verweij, 2021). CFI values greater than .95 indicate a good fit and values greater than .90 are acceptable (Hu & Bentler, 1999).

**Factor analysis supplemental results**

Full Factor Analysis Script can be found at [lone-GenSEM-MR/GenSEM Factor Analysis and GSEM.md at main · ellenmartin11/lone-GenSEM-MR (github.com)](https://github.com/ellenmartin11/lone-GenSEM-MR/blob/main/Analysis/GenSEM%20Factor%20Analysis%20and%20GSEM.md). This script also contains factor loadings for the 1 – 4 factor solutions.

***Scree Plot and Kaiser Criterion***

Supplementary Figure 1. *Scree Plot and Parallel Analysis to determine factor retention*


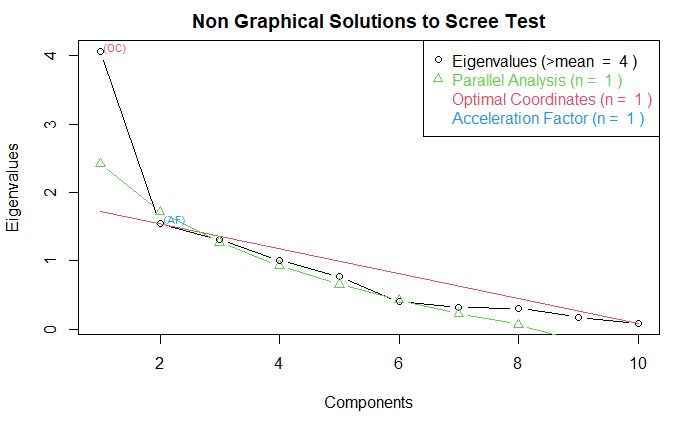


***Confirmatory Factor Analysis output***

CFA was specified on for the several factor solutions ranging from 1 – 4 factors.

**1 factor**

Supplementary Table 2*a. CFA output of the rejected 1 factor solution*


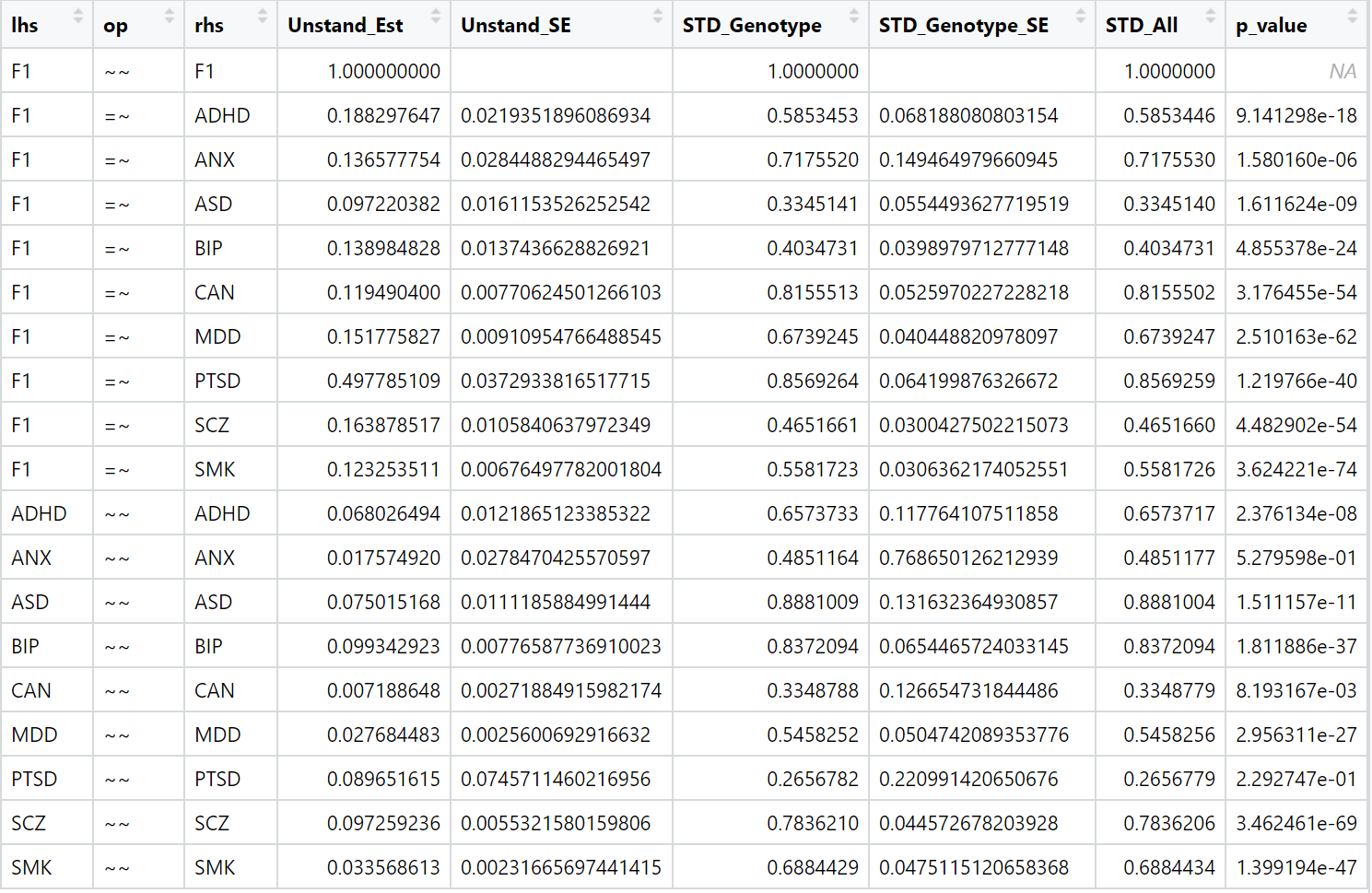


*Note.* SRMR = 0.15, CFI = 0.76

**2 factor**

F1 =~ ADHD + CAN + PTSD + SMK + MDD

F2 =~ ANX + BIP + MDD + SCZ

F1 ~~ F2

Supplementary Table 2*b. CFA output of the rejected 2 factor solution*


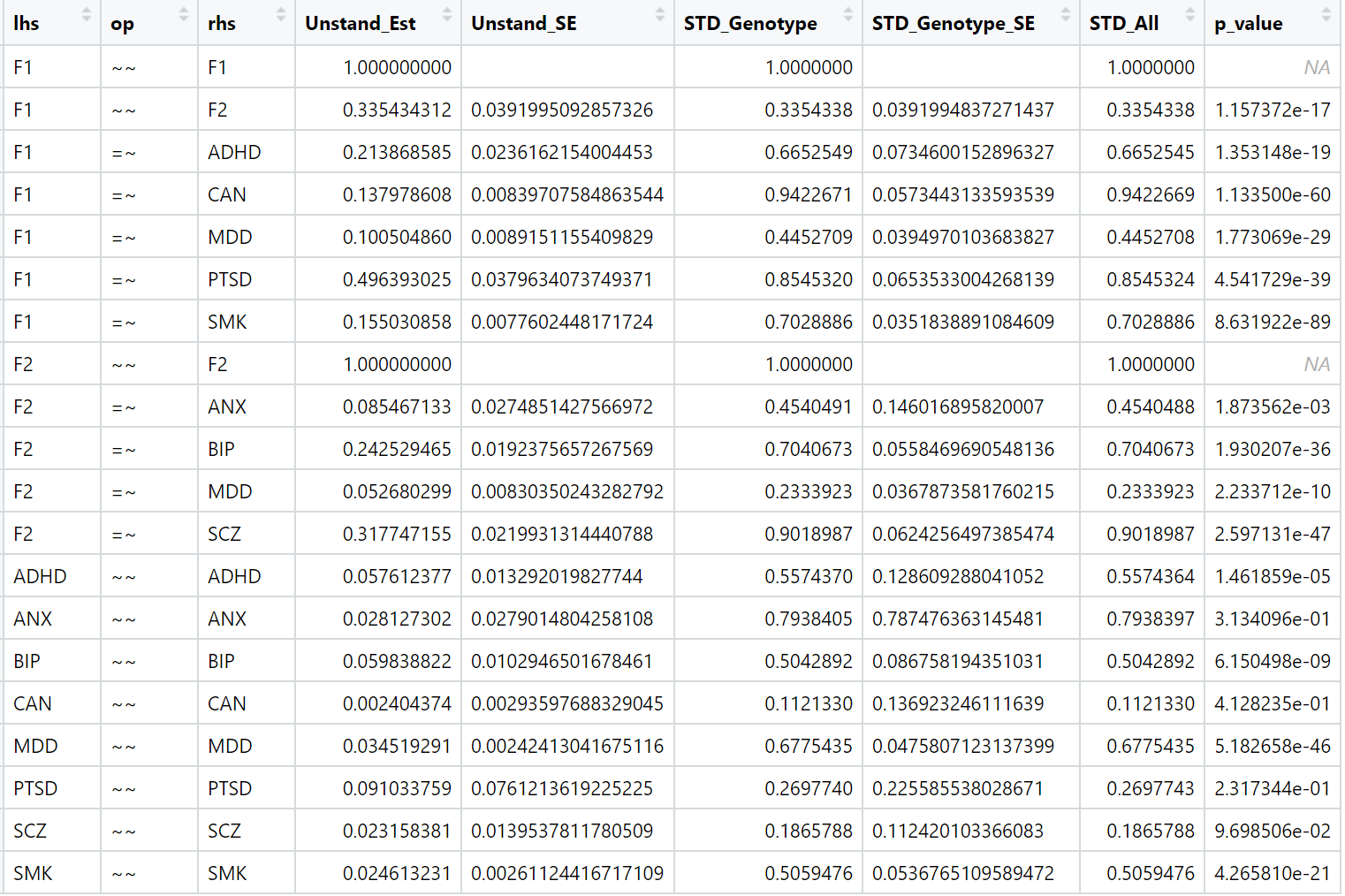


*Note.* SRMR = 0.19, CFI = 0.88

**3 factor – best fitting**

F1 =~ ADHD + ANX + ASD + MDD + PTSD

F2 =~ ALC + CAN + SMK

F3 =~ BIP + SCZ

F1~~F2

F1~~F3

F2~~F3

CAN~~a*CAN

SCZ~~a*SCZ

a > .001

*Note.* CAN and SCZ were constrained to be > .001 due to negative path estimates. SMK was forced to load on one factor rather than two for the sake of parsimony. Based on strong theoretical grounds, SMK was forced to load alongside the other two substance use phenotypes.

Supplementary Table 2*c. CFA output for the selected, best-fitting 3 factor solution used for GSEM*


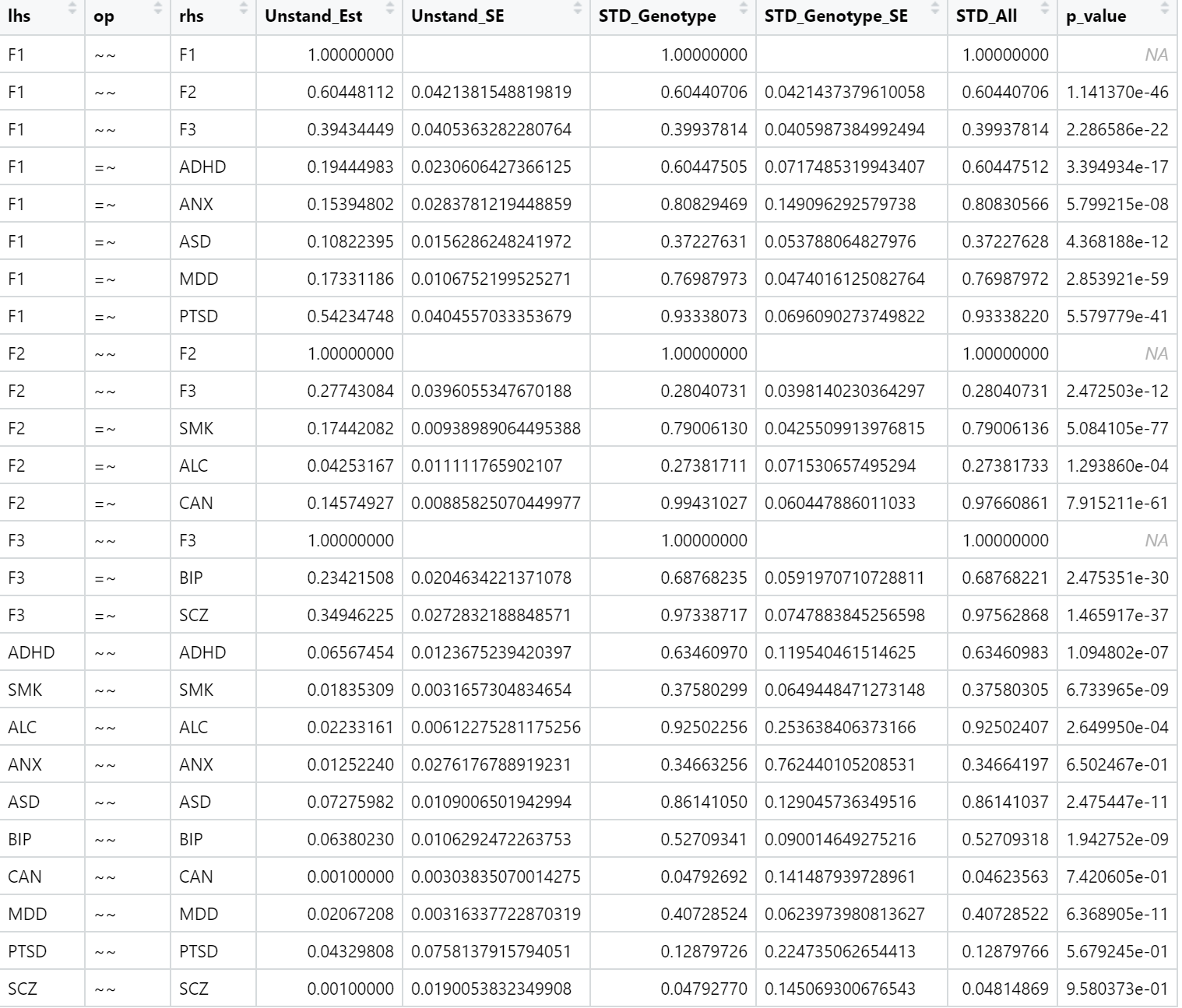


*Note.* SRMR = 0.11, CFI = 0.93

**3 factor – smoking on two factors**

F1 =~ NA*ADHD + ANX + ASD + MDD + PTSD + SMK

F2 =~ NA*ALC + CAN + SMK

F3 =~ NA*BIP + SCZ

F1~~F2

F1~~F3

F2~~F3

CAN~~a*CAN

a > .001

*Note.* CAN was constrained to avoid negative estimates. This model had unacceptable fit statistics.

Supplementary Table 2*d. CFA output for the rejected 3 factor alternate solution*


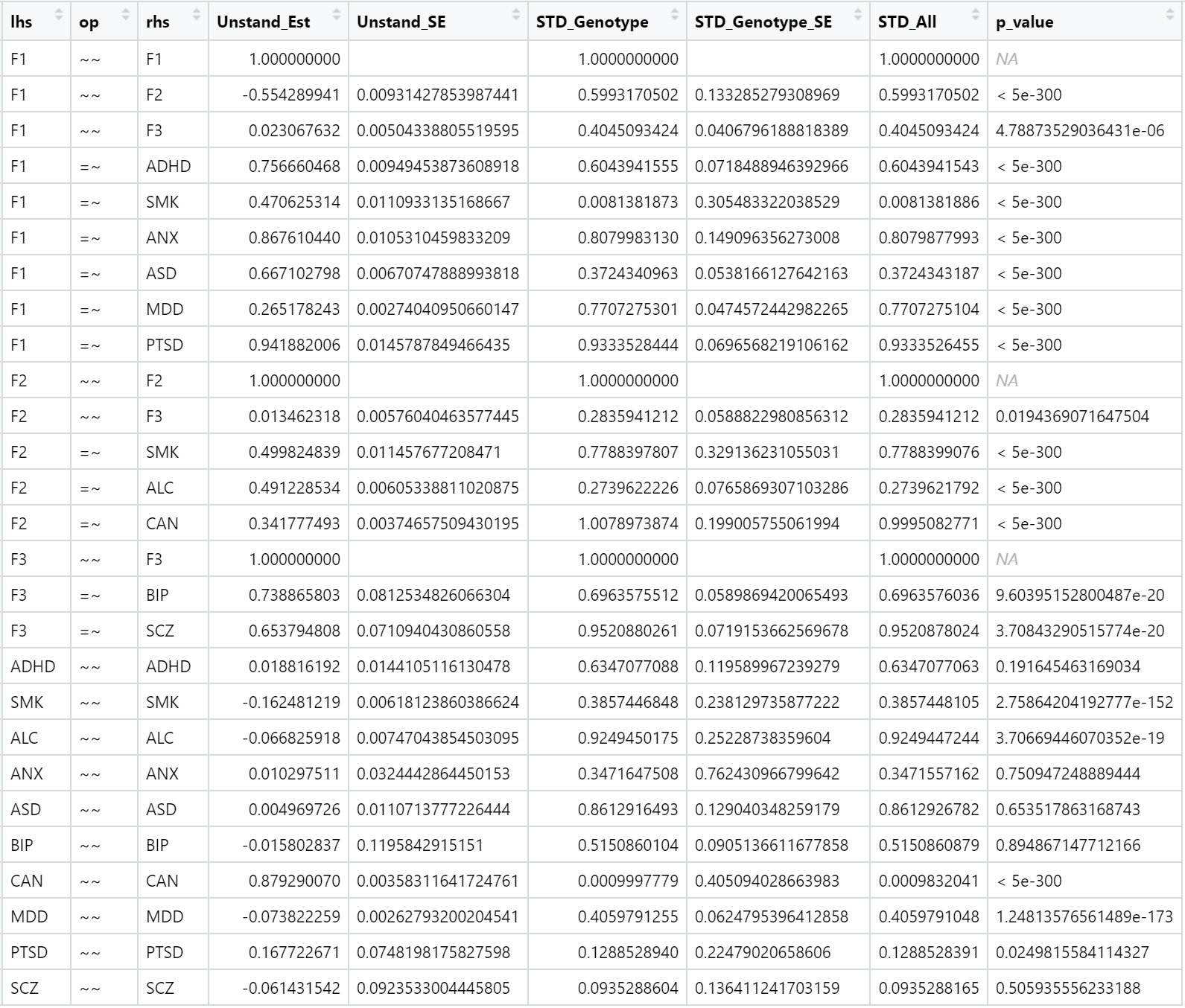


*Note.* SRMR = 7.62, CFI = -770

**4 factor**

F1 =~ ADHD + ASD + MDD + PTSD

F2 =~ ANX + MDD

F3 =~ BIP + SCZ

F4 =~ ALC + CAN + SMK

F1 ~~ F2

F1 ~~ F4

F1 ~~ F3

F2 ~~ F3

F2 ~~ F4

F3 ~~ F4

Supplementary Table 2*e. CFA output for the rejected 4 factor solution*


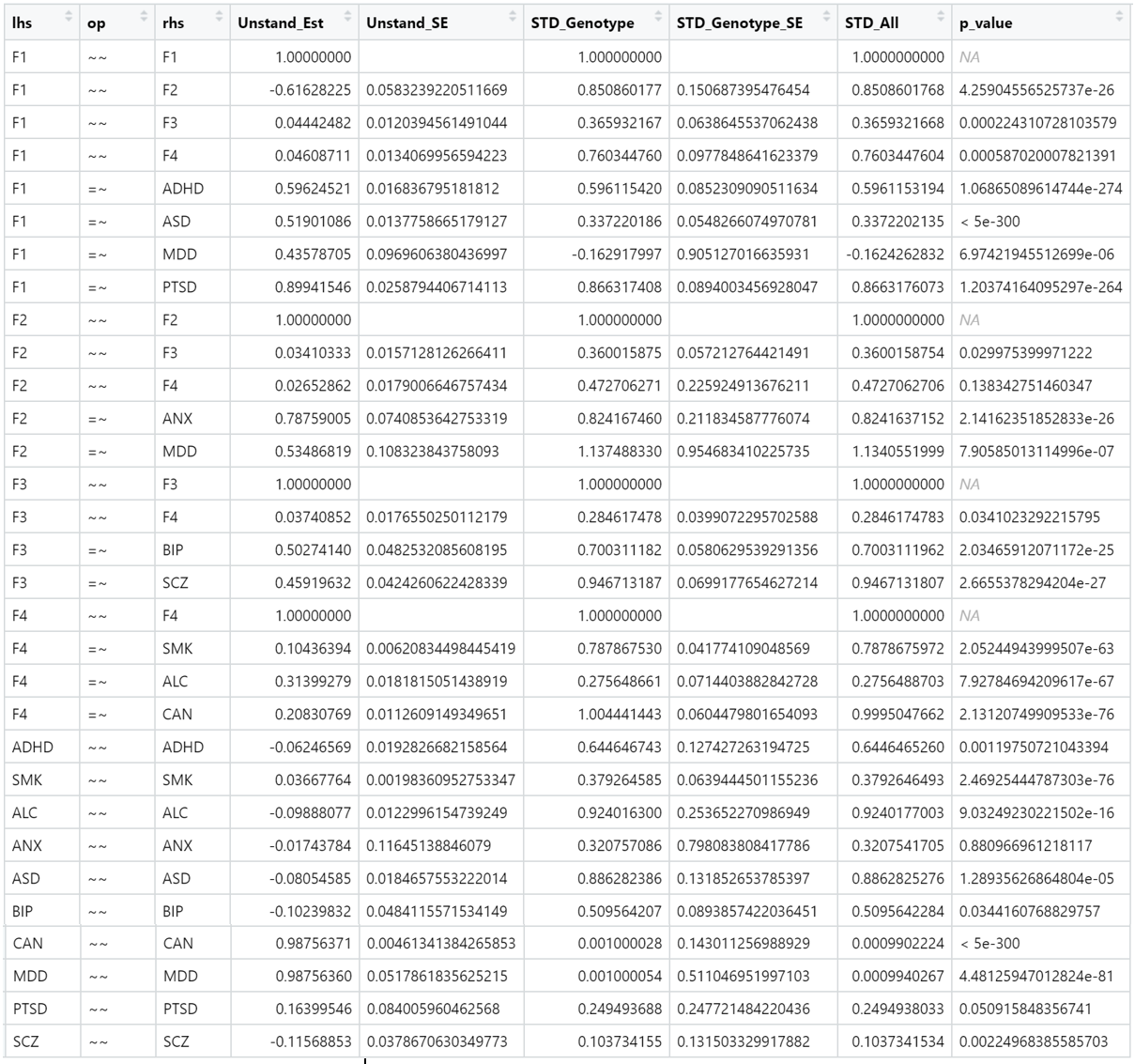


*Note.* SRMR = 7.48, CFI = -612

**S4 GENOMIC STRUCTURAL EQUATION MODEL SPECIFICATION**

The script used to execute GSEM can be found at [lone-GenSEM-MR/GenSEM Factor Analysis and GSEM.md at main · ellenmartin11/lone-GenSEM-MR (github.com)](https://github.com/ellenmartin11/lone-GenSEM-MR/blob/main/Analysis/GenSEM%20Factor%20Analysis%20and%20GSEM.md).

**Description of GSEM models**

Models were specified using the complete LDSC output (ODD and EVEN chromosomes combined).

In the first model, separate genetic correlations between loneliness and the latent factors were specified, thereby providing coefficient estimates that do not adjust for the genetic intercorrelations between the latent factors. Loneliness was specified as a latent factor due to syntactic requirements of *lavaan* (Rosseel, 2012).

In the second model, intercorrelations between F1 (NMD; neurodevelopmental and mood disorders), F2 (SUT; substance use traits) and F3 (DPF; disorders with psychotic features) were specified, statistically adjusting correlations between loneliness and the latent factors. Again, loneliness was specified as a latent factor.

The third model, which formed the basis of the later GWA and MR analyses, was the multivariate adjusted model with constraints applied to F2 (SUT) and F3 (DPF), forcing them to be 0 rather than negative. This was to check for potential over-inflation of the partial regression coefficient between LONE and F1.

To evaluate the strength of genetic associations, the standardised *beta* (β) regression coefficients were taken from the *STD_All* column in the *usermodel()*, a column that fully standardises both latent variables and observed variables. Confidence Intervals (CI’s) for all coefficients were computed using the standard error and standardised regression coefficients from the GSEM output columns, using the formula below.

$$\mathrm{lower} 95\% \mathrm{CI} = \beta- 1.96*\mathrm{SE}$$

$$upper 95\% \mathrm{CI} = \beta+ 1.96*\mathrm{SE}$$

***Unadjusted model specification***

unadjusted_model<- 'F1 =~ NA*ADHD + ANX + ASD + PTSD + MDD

F2 =~ NA*ALC + CAN + SMK

F3 =~ NA*BIP + SCZ

#latent indicator for loneliness

LONE =~ LONELINESS

LONE~~1*LONE

LONELINESS~~0*LONELINESS

#association with LONE

LONE ~~ F1 + F2 + F3’

Supplementary Figure 2*a. Path Diagram of the Unadjusted Model*


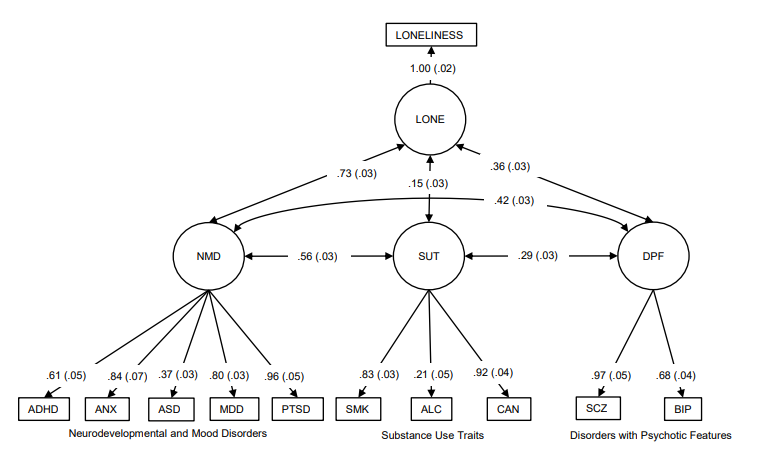


***Adjusted multivariate model specification***

#multivariate (adjusted), constrained model

Multivar <- '

#specifying factor structure

F1 =~ NA*ADHD + ANX + ASD + PTSD + MDD

F2 =~ NA*ALC + CAN + SMK

F3 =~ NA*BIP + SCZ

#latent indicator for loneliness

LONE =~ LONELINESS

LONE~~1*LONE

LONELINESS~~0*LONELINESS

#association with LONE

LONE ~ F1 + F2 + F3

#intercorrelations

F1 ~~ F2 + F3

F2 ~~ F3

Supplementary Figure 2*b. Path Diagram of the Adjusted Multivariate Model (Unconstrained)*


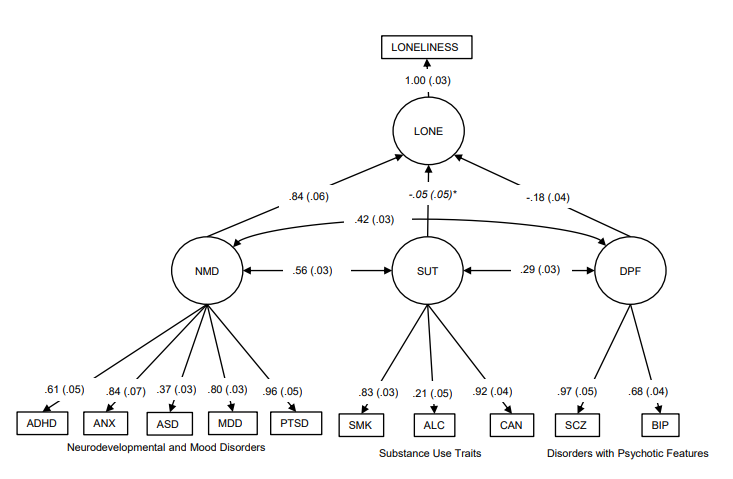


*Note.* The asterisk indicates statistical non-significance (*p* > .05)

***Final model (adjusted multivariate constrained model) specification***

*Note.* This model is shown in the main manuscript.

multivar_constrained<- 'F1 =~ NA*ADHD + ANX + ASD + PTSD + MDD

F2 =~ NA*ALC + CAN + SMK

F3 =~ NA*BIP + SCZ

#latent indicator for loneliness

LONE =~ LONELINESS

LONE~~1*LONE

LONELINESS~~0*LONELINESS

#with constraints

LONE ~ F1 + 0*F2 + 0*F3

#intercorrelations

F1 ~~ F2 + F3

F2 ~~ F3'

Supplementary Table 3. *Coefficients for the Final GSEM Model*


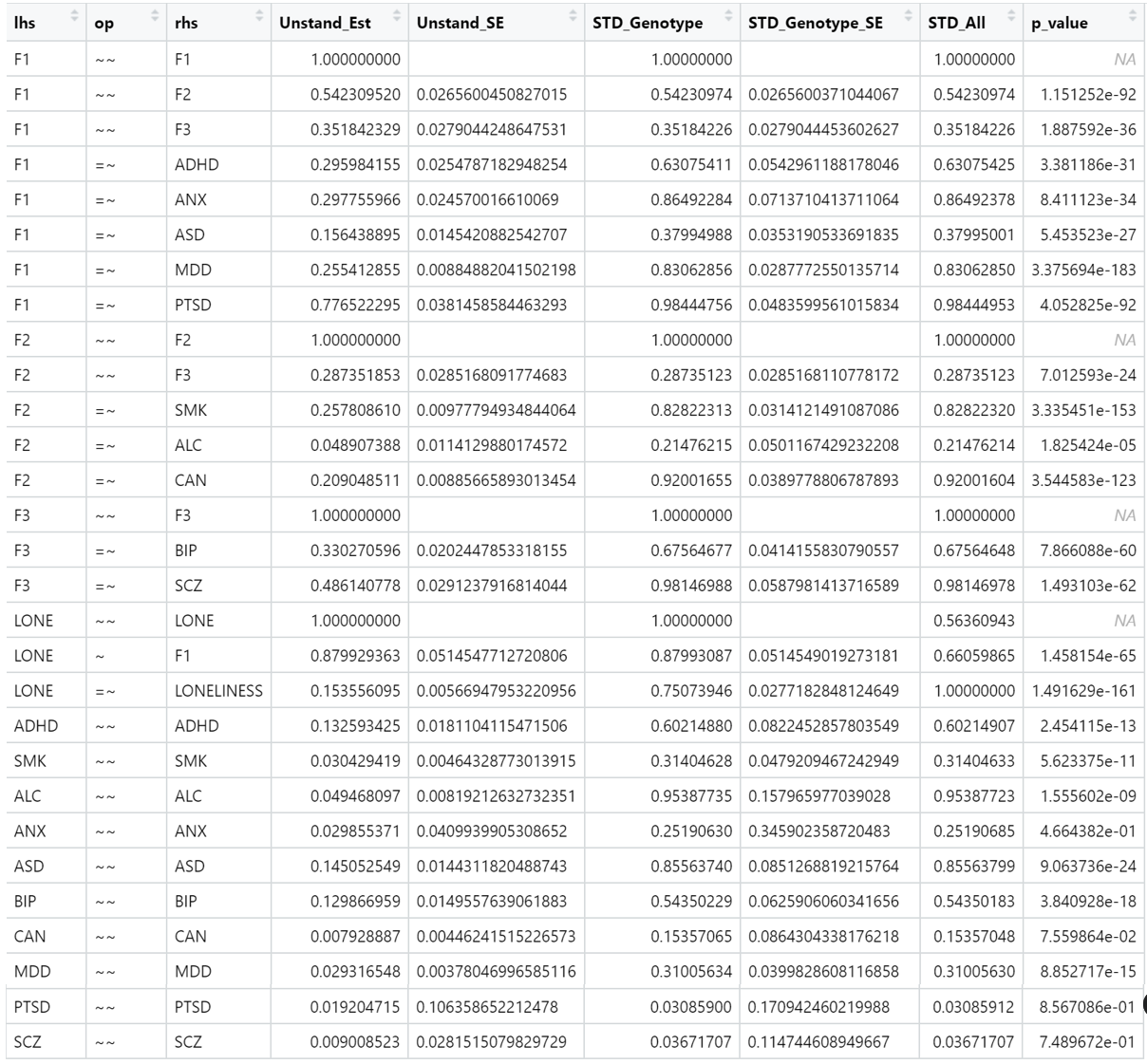


**S5 MULTIVARIATE GWAS**

**Desription of F1/NMD multivariate GWA**

Multivariate GWA was conducted on the entire GSEM model, but only the SNP effects related to NMD were retained due to the substantial genetic overlap observed between NMD and loneliness through GSEM. There were 22 LD-independent genome-wide SNPs for NMD. A model to compute heterogeneity statistics (Q) was also specified for later MR analyses. The online FUMA resource (Watanabe, Taskesen, van Bochoven, & Posthuma, 2017) was used for quick sanity checks of the GWA output. We specifically checked the lead SNPs to examine whether they have previously been linked to phenotypes that are related to neurodevelopmental/mood disorders. The lead SNPs have previously been associated with body-mass index, depression, sleep quality, schizophrenia, general risk tolerance and educational attainment. We also ensured that the SNPs were indeed expressed in the brain. Full results from FUMA can be found and downloaded at https://fuma.ctglab.nl/browse/500.

The script used to carry out multivariate GWA, including model specification, can be found at [lone-GenSEM-MR/GenSEMcluster.sh at main · ellenmartin11/lone-GenSEM-MR (github.com)](https://github.com/ellenmartin11/lone-GenSEM-MR/blob/main/Analysis/GenSEMcluster.sh). A manhattan plot was generated and can be seen below as well as a Q-Q plot.

Supplementary Figure 3*a. F1/NMD GWA Manhattan Plot*


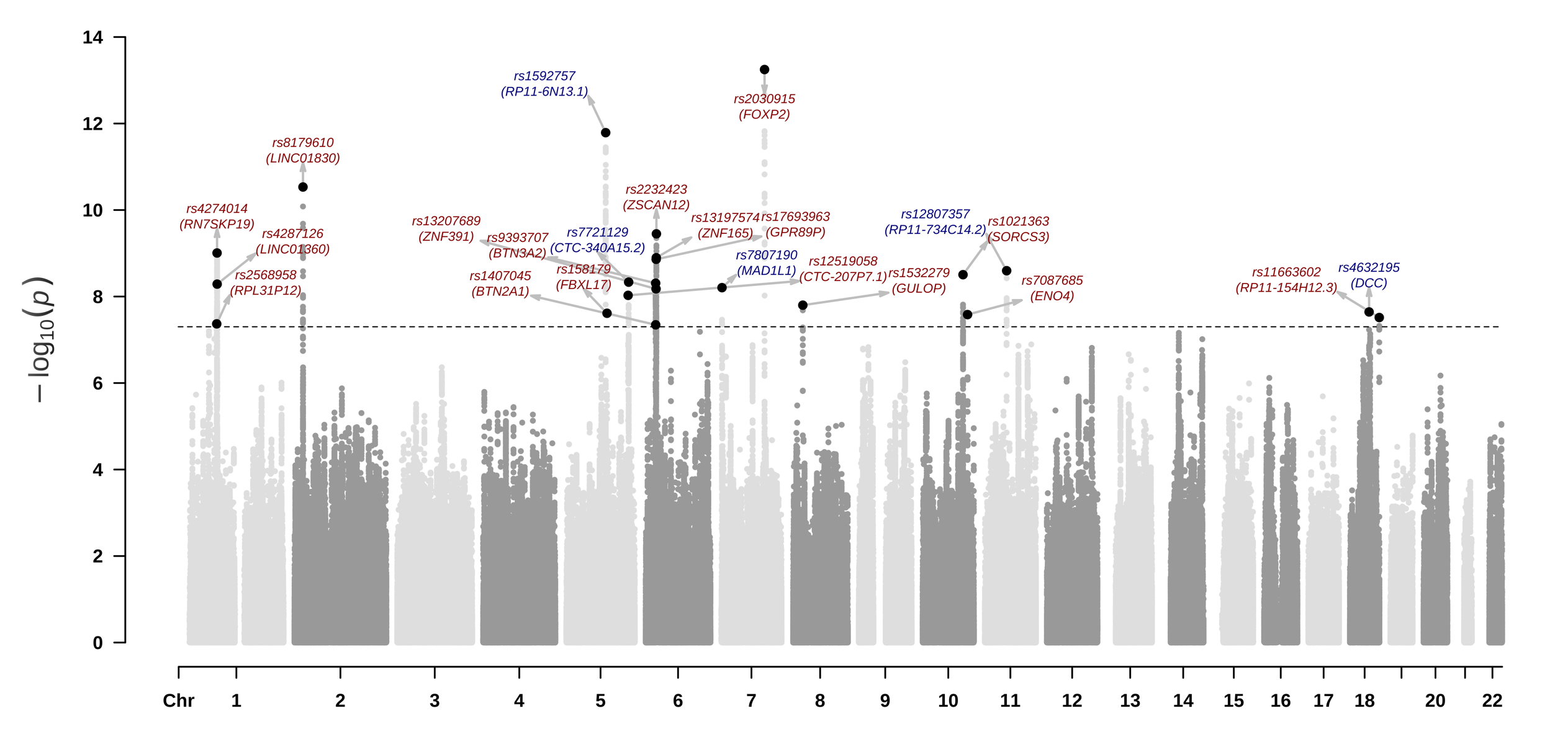


Supplementary Figure 3*b. Q-Q Plot of F1/NMD GWA*


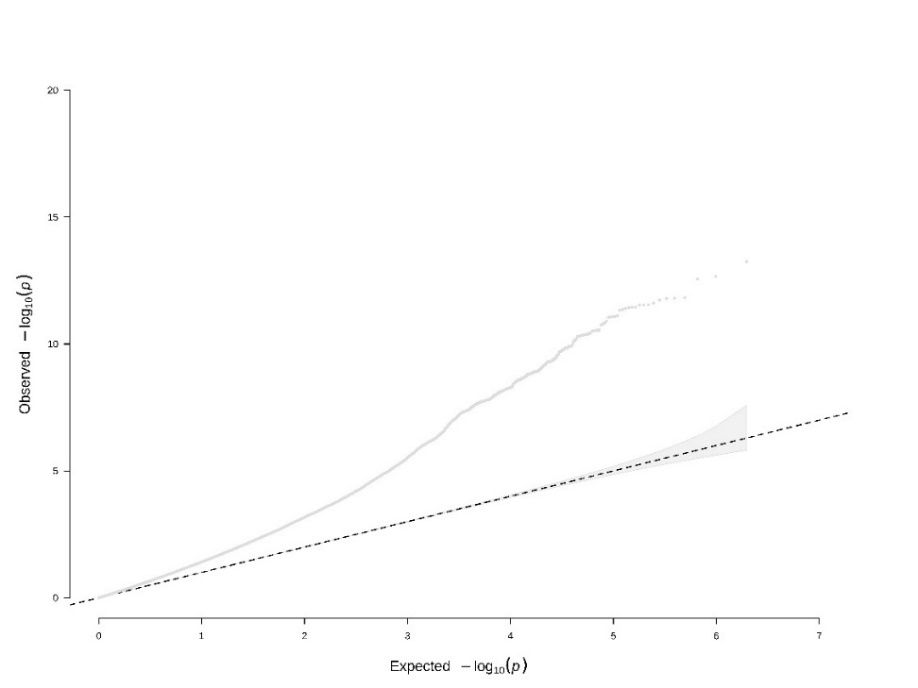


**Multivariate GWA summary statistics**

Supplementary Table 4. *Brief Summary of F1/NMD and Loneliness GWAS*

| GWAS | N (sample) | included SNPs | number of LD-independent genome-wide SNPs | smallest p-value | SNPs (shared) | SNPs (non-shared) |
| --- | --- | --- | --- | --- | --- | --- |
| F1 | 378372 | 1,968,630 | 22 | 5.6e-14 | 5 | 17 |
| Loneliness | 445024 | 1,968,630 | 18 | 3.8e-17 | 18 | 0 |

*Note.* SNPs (shared) refers to the number of genome-wide significant SNPs present in the other GWAS. *i.e.,* Of the 22 genome-wide significant SNPs in NMD, 5 are also present in the Loneliness GWA while the remaining 17 are not present in the Loneliness GWA. All 18 genome-wide SNPs in the Loneliness GWA are present in NMD.

**S6 BIDIRECTIONAL MENDELIAN RANDOMIZATION SUPPLEMENT**

**Description of Main MR methodology**

The full MR method can be found at [lone-GenSEM-MR/Mendelian Randomisation.md at main · ellenmartin11/lone-GenSEM-MR (github.com)](https://github.com/ellenmartin11/lone-GenSEM-MR/blob/main/Analysis/Mendelian%20Randomisation.md). The TwoSampleMR package by Hemani et al. (2017) was used.

1. Selection of genome-wide significant SNPs in the exposure (loneliness)
2. Harmonising SNPs across exposure and outcome (NMD)
3. Clumping (10,000 kb window, r2 < 0.001)
4. MR Analysis
   1. IVW and Sensitivity Analyses
   2. Test for Heterogeneity
   3. Test for Horizontal Pleiotropy
   4. Egger Sensitivity Test (more details available at GitHub)
   5. Steiger Filtering
5. Repeat steps using NMD (Q_SNP_ filtered) as the exposure and loneliness as the outcome for bidirectional analyses
6. Repeat steps specifying a less stringent p-value (p < 5 x 10^-07^) to increase the number of instruments
7. MR RAPS (Robust Adjusted Profile Score) for reduced p-value threshold

**Mendelian Randomization Loneliness to F1/NMD**

***Sensitivity analyses***

Supplementary Table 5*a. Heterogeneity Tests*

| outcome | exposure | method | Q | Q degrees of freedom | *p* |
| --- | --- | --- | --- | --- | --- |
| NMD | loneliness | MR Egger | 8.840471 | 9 | 0.4521287 |
| NMD | loneliness | Inverse variance weighted | 8.872238 | 10 | 0.5442702 |

Supplementary Table 5*b.* *Test of Directional Horizontal Pleiotropy*

| outcome | exposure |  | Egger intercept | SE | *p* |
| --- | --- | --- | --- | --- | --- |
| NMD | loneliness |  | -0.0007018 | 0.0039373 | 0.8624872 |

*Egger Sensitivity Test*

I2 = .972

Supplementary Table 5*c. Steiger Test of Directionality*

| exposure | outcome | exposure SNP *r*^2^ | outcome SNP *r*^2^ | Correct causal direction | *p* |
| --- | --- | --- | --- | --- | --- |
| loneliness | NMD | 0.0015155 | 0.0002397 | TRUE | 4.87 x 10^-13^ |

*Note.* All SNPS were found to be in the correct causal direction.

***Scatter plot***

Supplementary Figure 4*a. Scatter Plot of Loneliness to F1/NMD*


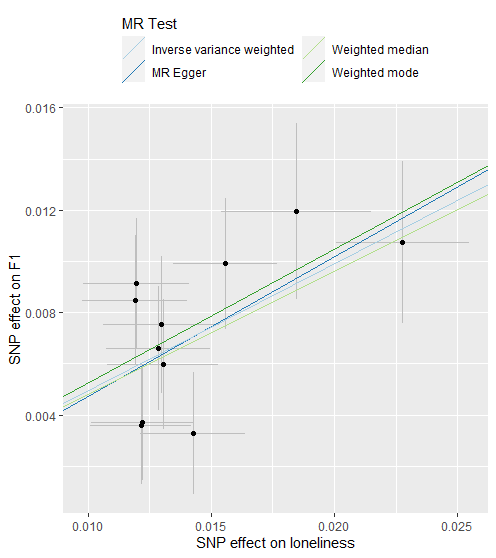


Supplementary Table 5*d. NMD* *SNPs used as genetic instruments for Mendelian Randomization*

| GWA data | SNP (annotated gene) | A1 | A2 | BETA | SE | P (GWA) | P (chi-square) | P (chi-square, dichotomized) | consequence | description |
| --- | --- | --- | --- | --- | --- | --- | --- | --- | --- | --- |
| NMD | rs2030915 (FOXP2) | C | G | 0.018 | 0.002 | 5.65E-14 | 0 | Q_sig | intron | forkhead box P2 [Source:HGNC Symbol;Acc:HGNC:13875] |
|  | rs1592757 (RP11-6N13.1) | G | C | -0.022 | 0.003 | 1.63E-12 | 3.93e-07 | Q_ns | intron |  |
|  | rs8179610 (LINC01830) | G | T | -0.015 | 0.002 | 2.94E-11 | 0 | Q_sig | intron | long intergenic non-protein coding RNA 1830 [Source:HGNC Symbol;Acc:HGNC:52636] |
|  | rs2232423 (ZSCAN12) | A | G | 0.028 | 0.004 | 3.55E-10 | 6e-81 | Q_sig | missense | zinc finger and SCAN domain containing 12 [Source:HGNC Symbol;Acc:HGNC:13172] |
|  | rs4274014 (RN7SKP19) | C | A | -0.017 | 0.003 | 9.85E-10 | 1e-21 | Q_sig | intergenic | RN7SK pseudogene 19 [Source:HGNC Symbol;Acc:HGNC:45743] |
|  | rs13197574 (ZNF165) | T | C | 0.027 | 0.004 | 1.27E-09 | 4.94e-78 | Q_sig | downstream | zinc finger protein 165 [Source:HGNC Symbol;Acc:HGNC:12953] |
|  | rs17693963 (GPR89P) | A | C | 0.026 | 0.004 | 1.38E-09 | 1.58e-68 | Q_sig | upstream | G protein-coupled receptor 89 pseudogene [Source:HGNC Symbol;Acc:HGNC:13951] |
|  | rs12807357 (RP11-734C14.2) | C | T | -0.017 | 0.003 | 2.53E-09 | 0.000364 | Q_ns | intergenic |  |
|  | rs1021363 (SORCS3) | A | G | 0.017 | 0.003 | 3.13E-09 | 1.41e-20 | Q_sig | intron | sortilin related VPS10 domain containing receptor 3 [Source:HGNC Symbol;Acc:HGNC:16699] |
|  | rs7721129 (CTC-340A15.2) | G | A | -0.015 | 0.003 | 4.64E-09 | 0.999 | Q_ns | intron |  |
|  | rs9393707 (BTN3A2) | T | C | 0.023 | 0.004 | 4.92E-09 | 1.37e-41 | Q_sig | upstream | butyrophilin subfamily 3 member A2 [Source:HGNC Symbol;Acc:HGNC:1139] |
|  | rs4287126 (LINC01360) | T | C | 0.016 | 0.003 | 5.16E-09 | 9.42e-26 | Q_sig | intergenic | long intergenic non-protein coding RNA 1360 [Source:HGNC Symbol;Acc:HGNC:50593] |
|  | rs7807190 (MAD1L1) | A | G | 0.018 | 0.003 | 6.23E-09 | 1 | Q_ns | downstream | mitotic arrest deficient 1 like 1 [Source:HGNC Symbol;Acc:HGNC:6762] |
|  | rs13207689 (ZNF391) | C | G | 0.025 | 0.004 | 6.66E-09 | 7.2e-75 | Q_sig | 3_prime_UTR | zinc finger protein 391 [Source:HGNC Symbol;Acc:HGNC:18779] |
|  | rs12519058 (CTC-207P7.1) | G | A | -0.011 | 0.002 | 9.39E-09 | 0 | Q_sig | intergenic |  |
|  | rs1532279 (GULOP) | C | G | 0.012 | 0.002 | 1.58E-08 | 0 | Q_sig | intron | gulonolactone (L-) oxidase, pseudogene [Source:HGNC Symbol;Acc:HGNC:4695] |
|  | rs4632195 (DCC) | C | T | -0.015 | 0.003 | 2.27E-08 | 1.45e-07 | Q_ns | intron | DCC netrin 1 receptor [Source:HGNC Symbol;Acc:HGNC:2701] |
|  | rs158179 (FBXL17) | T | A | 0.014 | 0.002 | 2.44E-08 | 3.59e-303 | Q_sig | intron | F-box and leucine rich repeat protein 17 [Source:HGNC Symbol;Acc:HGNC:13615] |
|  | rs7087685 (ENO4) | G | A | -0.013 | 0.002 | 2.62E-08 | 0 | Q_sig | intron | enolase 4 [Source:HGNC Symbol;Acc:HGNC:31670] |
|  | rs11663602 (RP11-154H12.3) | C | A | -0.016 | 0.003 | 3.06E-08 | 1.54e-12 | Q_sig | downstream |  |
|  | rs2568958 (RPL31P12) | G | A | -0.014 | 0.003 | 4.28E-08 | 1.67e-35 | Q_sig | upstream | ribosomal protein L31 pseudogene 12 [Source:HGNC Symbol;Acc:HGNC:35546] |
|  | rs1407045 (BTN2A1) | A | G | -0.012 | 0.002 | 4.51E-08 | 1.29e-273 | Q_sig | intron | butyrophilin subfamily 2 member A1 [Source:HGNC Symbol;Acc:HGNC:1136] |

Supplementary Table 5*e. Loneliness SNPs used as genetic instruments for Mendelian Randomization*

| Loneliness | rs613872 (TCF4) | G | T | -0.023 | 0.003 | 3.76E-17 | 1 | Q_ns | intron | transcription factor 4 [Source:HGNC Symbol;Acc:HGNC:11634] |
| --- | --- | --- | --- | --- | --- | --- | --- | --- | --- | --- |
|  | rs7044244 (PHF2) | G | A | 0.016 | 0.002 | 1.32E-13 | 1 | Q_ns | intron | PHD finger protein 2 [Source:HGNC Symbol;Acc:HGNC:8920] |
|  | rs2069117 (LINC01470) | A | C | -0.014 | 0.002 | 1.92E-11 | 0.0107 | Q_ns | intron | long intergenic non-protein coding RNA 1470 [Source:HGNC Symbol;Acc:HGNC:51105] |
|  | rs1532896 (RP11-397A16.3) | C | T | -0.016 | 0.003 | 5.09E-10 | 1 | Q_ns | upstream |  |
|  | rs11867618 (BPTF) | G | A | -0.018 | 0.003 | 1.20E-09 | 1 | Q_ns | intron | bromodomain PHD finger transcription factor [Source:HGNC Symbol;Acc:HGNC:3581] |
|  | rs9388863 (EPB41L2) | T | G | -0.013 | 0.002 | 1.25E-09 | 1 | Q_ns | intron | erythrocyte membrane protein band 4.1 like 2 [Source:HGNC Symbol;Acc:HGNC:3379] |
|  | rs2273146 (DDX27) | C | T | 0.012 | 0.002 | 3.37E-09 | 0.00122 | Q_ns | intron | DEAD-box helicase 27 [Source:HGNC Symbol;Acc:HGNC:15837] |
|  | rs4462992 (HSPE1P19) | T | C | 0.012 | 0.002 | 3.50E-09 | 1 | Q_ns | intergenic | heat shock protein family E (Hsp10) member 1 pseudogene 19 [Source:HGNC Symbol;Acc:HGNC:49338] |
|  | rs2126787 (RP11-397A16.1) | G | A | -0.016 | 0.003 | 3.76E-09 | 1 | Q_ns | intergenic |  |
|  | rs1261070 (TCF4) | A | G | -0.021 | 0.004 | 4.56E-09 | 1 | Q_ns | intron | transcription factor 4 [Source:HGNC Symbol;Acc:HGNC:11634] |
|  | rs7737302 (CTB-95D12.1) | C | T | 0.013 | 0.002 | 7.77E-09 | 1 | Q_ns | intergenic |  |
|  | rs1966836 (OR1S1) | A | G | 0.013 | 0.002 | 7.90E-09 | 1 | Q_ns | missense | olfactory receptor family 1 subfamily S member 1 [Source:HGNC Symbol;Acc:HGNC:8227] |
|  | rs1997851 (CSE1L) | T | G | -0.012 | 0.002 | 1.18E-08 | 0.112 | Q_ns | intergenic | chromosome segregation 1 like [Source:HGNC Symbol;Acc:HGNC:2431] |
|  | rs1439252 (ERBB4) | G | A | 0.012 | 0.002 | 1.94E-08 | 1 | Q_ns | intron | erb-b2 receptor tyrosine kinase 4 [Source:HGNC Symbol;Acc:HGNC:3432] |
|  | rs7105282 (NUP160) | C | A | 0.012 | 0.002 | 2.55E-08 | 1 | Q_ns | intron | nucleoporin 160 [Source:HGNC Symbol;Acc:HGNC:18017] |
|  | rs17453775 (CTB-95D12.1) | C | T | 0.013 | 0.002 | 3.07E-08 | 2.03e-06 | Q_ns | intergenic |  |
|  | rs495146 (NA) | C | T | -0.013 | 0.002 | 4.14E-08 | 0.0112 | Q_ns | - |  |
|  | rs1317149 (CELF1) | C | T | 0.012 | 0.002 | 4.81E-08 | 0.999 | Q_ns | downstream | CUGBP Elav-like family member 1 [Source:HGNC Symbol;Acc:HGNC:2549] |

**Mendelian Randomization NMD (Q_SNP_ filtered) to Loneliness**

***Sensitivity Analyses***

Supplementary Table *5f. Heterogeneity Tests*

| outcome | exposure | method | Q | Q degrees of freedom | *p* |
| --- | --- | --- | --- | --- | --- |
| loneliness | NMD | MR Egger | 20.81972 | 8 | 7.64 x 10^-03^ |
| loneliness | NMD | Inverse variance weighted | 21.15064 | 9 | 1.20 x 10^-02^ |

Supplementary Table *5g. Test of Directional Horizontal Pleiotropy*

| outcome | exposure | Egger intercept | SE | *p* |
| --- | --- | --- | --- | --- |
| loneliness | NMD | -0.002 | 0.0049295 | 7.31 x 10^-01^ |

*Egger Sensitivity Test*

I2 = .971

Supplementary Table 5*h*. *Steiger Test of Directionality*

| exposure | outcome | exposure SNP *r*^2^ | outcome SNP *r*^2^ | Correct causal direction | *p* |
| --- | --- | --- | --- | --- | --- |
| NMD | loneliness | 0.0011072 | 0.0002447 | TRUE | 1.49 x 10^-15^ |

*Note.* All SNPs were found to be in the correct direction

***Scatter plot***

Supplementary Figure 4*b. Scatter Plot of F1/NMD to Loneliness*


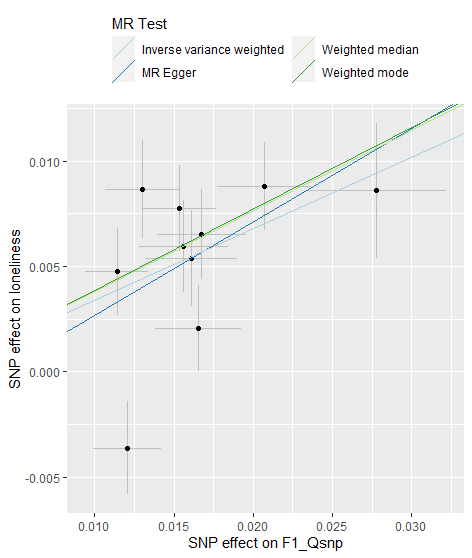


***Forest plot***

Supplementary Figure 4*c. Forest Plot of F1/NMD to Loneliness*


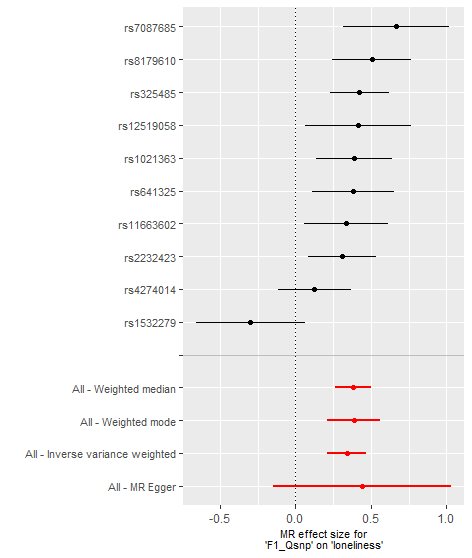


**Mendelian Randomization Loneliness to NMD (p < 5x10^-07^)**

***Main MR Analyses***

Supplementary Table 5*i. Table of MR Methods*

| outcome | exposure | method | nsnp | *b* | SE | *p* |
| --- | --- | --- | --- | --- | --- | --- |
| NMD | Loneliness_5e-7 | MR Egger | 24 | 0.2901209 | 0.3827217 | 4.56x10^-01^ |
| NMD | Loneliness_5e-7 | Inverse variance weighted | 24 | 0.4898733 | 0.0741826 | 4.01x10^-11^ |
| NMD | Loneliness_5e-7 | Weighted mode | 24 | 0.5341551 | 0.1055374 | 4.01x10^-05^ |
| NMD | Loneliness_5e-7 | Weighted median | 24 | 0.4828668 | 0.0623770 | 9.86x10^-15^ |

***Sensitivity Analyses***

Supplementary Table 5*j. Heterogeneity Test*

| outcome | exposure | method | Q | Q degrees of freedom | *p* |
| --- | --- | --- | --- | --- | --- |
| NMD | Loneliness_5e-7 | MR Egger | 74.76671 | 22 | 1.00x10^-05^ |
| NMD | Loneliness_5e-7 | Inverse variance weighted | 75.72983 | 23 | 2.00x10^-07^ |

*Note.* There is evidence of heterogeneity.

Supplementary Table 5*k. Horizontal Pleiotropy Test*

| outcome | exposure | Egger intercept | SE | *p* |
| --- | --- | --- | --- | --- |
| NMD | Loneliness_5e-7 | 0.0026415 | 0.004962 | 0.5998197 |

*Egger Sensitivity Test*

I2 = .970

Supplementary Table 5*l. Steiger Test of Directionality*

| exposure | outcome | exposure SNP *r*^2^ | outcome SNP *r*^2^ | Correct causal direction | *p* |
| --- | --- | --- | --- | --- | --- |
| Loneliness_5e-7 | NMD | 0.0017882 | 0.0005796 | TRUE | 0 |

*Note.* 2 SNPs were found to be operating in the incorrect direction. However, results of MR Steiger are consistent with the pattern of findings in Supplementary Table 5*i*. Full results are available at [lone-GenSEM-MR/Bidirectional Mendelian Randomization Lone and F1.md at main · ellenmartin11/lone-GenSEM-MR (github.com)](https://github.com/ellenmartin11/lone-GenSEM-MR/blob/main/Results/Bidirectional%20Mendelian%20Randomization%20Lone%20and%20F1.md).

***Forest Plot***

Supplementary Figure 4*c. Forest Plot of Loneliness to NMD/F1 with the less stringent p-value threshold*


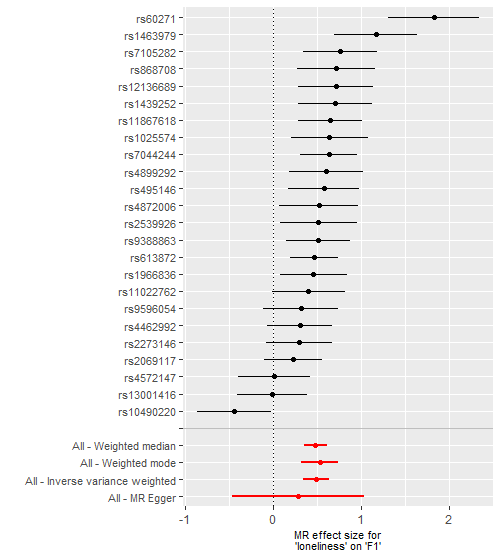


***Scatter Plot***

Supplementary Figure 4*d. Scatter plot of Loneliness to NMD/F1 with the less stringent p-value threshold*


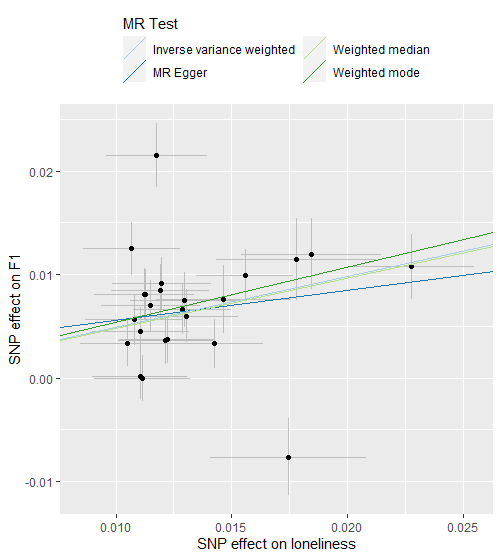


***MR RAPS***

Supplementary Table 5*m.* MR RAPS

| exposure | outcome | ^β | SE | *p* |
| --- | --- | --- | --- | --- |
| Loneliness_5e-7 | NMD | 0.5128462 | 0.06853184 | 7.24 x 10^-14^ |

**Mendelian Randomization NMD (Q_SNP_ filtered) to Loneliness (p < 5x10^-07^)**

***Main MR Analyses***

Supplementary Table 5*n. Table of MR Methods*

| outcome | exposure | method | nsnp | *b* | SE | *p* |
| --- | --- | --- | --- | --- | --- | --- |
| Loneliness | NMD_5e-7 | MR Egger | 19 | 0.2059939 | 0.2780956 | 4.69 x 10^-01^ |
| Loneliness | NMD_5e-7 | Inverse variance weighted | 19 | 0.3466251 | 0.0548549 | 2.63 x 10^-10^ |
| Loneliness | NMD_5e-7 | Weighted mode | 19 | 0.3661856 | 0.0769367 | 1.58 x 10^-04^ |
| Loneliness | NMD_5e-7 | Weighted median | 19 | 0.3358195 | 0.0520162 | 1.53 x 10^-10^ |

***Sensitivity Analyses***

Supplementary Table 5*o. Heterogeneity Test*

| outcome | exposure | method | Q | Q degrees of freedom | *p* |
| --- | --- | --- | --- | --- | --- |
| Loneliness | NMD_5e-7 | MR Egger | 48.75725 | 17 | 6.57 x 10^-05^ |
| Loneliness | NMD_5e-7 | Inverse variance weighted | 49.52169 | 18 | 8.91 x 10^-05^ |

*Note.* There is evidence of heterogeneity.

Supplementary Table 5*p. Horizontal Pleiotropy Test*

| outcome | exposure | Egger intercept | SE | *p* |
| --- | --- | --- | --- | --- |
| Loneliness | NMD_5e-7 | 0.0021538 | 0.0041719 | 6.12 x 10^-01^ |

*Egger Sensitivity Test*

I2 = .964

Supplementary Table 5*q. Steiger Test of Directionality*

| exposure | outcome | exposure SNP *r*^2^ | outcome SNP *r*^2^ | Correct causal direction | *p* |
| --- | --- | --- | --- | --- | --- |
| NMD_5e-7 | Loneliness | 0.0018235 | 0.000444 | TRUE | 0 |

*Note.* One instrument was found to be operating in the incorrect causal direction. MR Steiger was conducted using 18 instruments operating in the correct causal direction. The results are consistent with those shown in Supplementary Table 5*n* and can be found at the GitHub page.

***Forest Plot***

Supplementary Figure 4*e. Forest Plot*


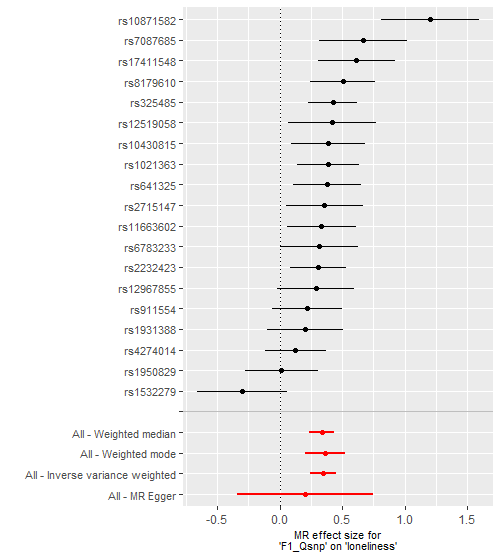


***Scatter Plot***

Supplementary Figure 4*f. Scatter Plot*


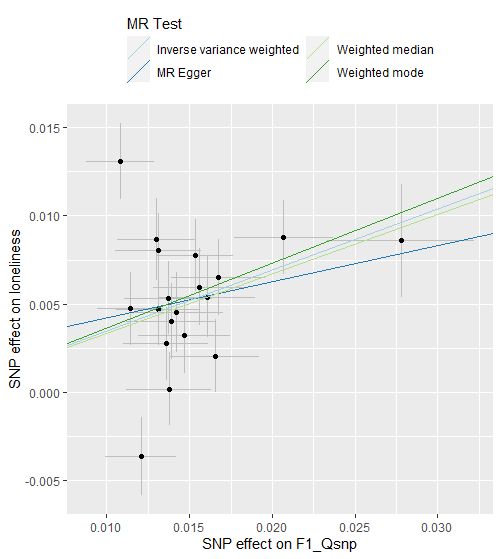


***MR RAPS***

Supplementary Table 5*r.* MR RAPS

| exposure | outcome | ^β | SE | *p* |
| --- | --- | --- | --- | --- |
| NMD_5e-7 | Loneliness | 0.358 | 0.0497 | 1.33 x 10^-15^ |

**S7 DISCUSSION SUPPLEMENT**

**Negative associations between loneliness and substance use and loneliness and disorders with psychotic features in the unconstrained multivariate adjusted model**

After controlling for the intercorrelations between the three latent factors, loneliness was negatively associated with DPF and was no longer significantly associated with SUT. These findings are consistent with Gao et al. (2016) polygenic score analyses which provided modest evidence of negative coheritability between loneliness and schizophrenia and between loneliness and bipolar disorder. This sign reversal is likely to be statistical phenomenon that occurs because of multicollinearity (Tu, Gunnell, & Gilthorpe, 2008). It would be difficult to interpret the meaning of negative genetic associations considering extensive evidence previously supporting the genetic correlation between loneliness and disorders with psychotic features and between loneliness and substance use. Therefore, our observations of (a) a statistically significant negative association between loneliness and disorders with psychotic features, and (b) a non-significant negative association between loneliness and substance use, may be better explained in other ways.

One possible explanation of our findings is that the overlap between loneliness and disorders with psychotic features (DPF) and between loneliness and substance use (SUT) may be better accounted for by other factors. Potential factors include stigmatisation, where substance users and those with psychotic experiences may be socially ostracised, but similarly, these individuals may process social cues in a distorted manner due to cognitive changes induced by substance use or the onset of psychotic features (Ingram et al., 2020; Leathem, Currin, Montoya, & Karlsgodt, 2021). Alternatively, loneliness and other social difficulties might encourage experimentation with substance (Copeland, Fisher, Moody, & Feinberg, 2018) and elevate the risk of psychotic symptoms (Heins et al., 2019).

Another possible explanation of our findings is that the association between loneliness and DPF be mediated through the association between loneliness and the neurodevelopmental/mood disorders latent factor (NMD). The same may be true for the association between loneliness and SUT. This explanation suggests that the co-occurrence of loneliness and disorders with psychotic features, and between loneliness and substance use traits, can be accounted for by the experience of mood problems that is common to those phenotypes. It would be beneficial to conduct further analyses such as specifying a mediation model to determine which of these explanations is the most likely.

Regardless, both explanations highlight the importance of investigating the relationship between loneliness and neurodevelopmental/mood disorders. The first explanation implies that loneliness and neurodevelopmental/mood disorders are genetically linked in a way that loneliness and disorders with psychotic features and loneliness and substance use are not. The second explanation suggests that neurodevelopmental/mood disorders may play a role in explaining the relationship between loneliness and psychotic disorders, as well as that between loneliness and substance use disorders. In both cases, a strong connection between loneliness and neurodevelopmental/mood disorders seems to be emphasised and re-iterates what existing literature has so far suggested.

**References**

Abdellaoui, A., Smit, D. J. A., van den Brink, W., Denys, D., & Verweij, K. J. H. (2021). Genomic relationships across psychiatric disorders including substance use disorders. *Drug and Alcohol Dependence*, *220*. https://doi.org/10.1016/J.DRUGALCDEP.2021.108535

Altshuler, D. M., Gibbs, R. A., Peltonen, L., Schaffner, S. F., Yu, F., Dermitzakis, E., … McEwen, J. E. (2010). Integrating common and rare genetic variation in diverse human populations. *Nature*, *467*(7311), 52. https://doi.org/10.1038/NATURE09298

Bulik-Sullivan, B., Finucane, H. K., Anttila, V., Gusev, A., Day, F. R., Loh, P. R., … Neale, B. M. (2015). An atlas of genetic correlations across human diseases and traits. *Nature Genetics*, *47*(11), 1236–1241. https://doi.org/10.1038/NG.3406

Copeland, M., Fisher, J. C., Moody, J., & Feinberg, M. E. (2018). Different Kinds of Lonely: Dimensions of Isolation and Substance Use in Adolescence. *Journal of Youth and Adolescence*, *47*(8), 1755–1770. https://doi.org/10.1007/S10964-018-0860-3/TABLES/10

Day, F. R., Ong, K. K., & Perry, J. R. B. (2018). Elucidating the genetic basis of social interaction and isolation. *Nature Communications 2018 9:1*, *9*(1), 1–6. https://doi.org/10.1038/s41467-018-04930-1

Demontis, D., Walters, R. K., Martin, J., Mattheisen, M., Als, T. D., Agerbo, E., … Neale, B. M. (2018). Discovery of the first genome-wide significant risk loci for attention deficit/hyperactivity disorder. *Nature Genetics 2018 51:1*, *51*(1), 63–75. https://doi.org/10.1038/s41588-018-0269-7

Finch, H. (2006). Comparison of the Performance of Varimax and Promax Rotations: Factor Structure Recovery for Dichotomous Items. *Journal of Educational Measurement*, *43*(1), 39–52. https://doi.org/10.1111/J.1745-3984.2006.00003.X

Forero, C. G., Maydeu-Olivares, A., & Gallardo-Pujol, D. (2009). Factor Analysis with Ordinal Indicators: A Monte Carlo Study Comparing DWLS and ULS Estimation. *Http://Dx.Doi.Org/10.1080/10705510903203573*, *16*(4), 625–641. https://doi.org/10.1080/10705510903203573

Gao, J., Davis, L. K., Hart, A. B., Sanchez-Roige, S., Han, L., Cacioppo, J. T., & Palmer, A. A. (2016). Genome-Wide Association Study of Loneliness Demonstrates a Role for Common Variation. *Neuropsychopharmacology 2017 42:4*, *42*(4), 811–821. https://doi.org/10.1038/npp.2016.197

Grove, J., Ripke, S., Als, T. D., Mattheisen, M., Walters, R. K., Won, H., … Børglum, A. D. (2019). Identification of common genetic risk variants for autism spectrum disorder. *Nature Genetics 2019 51:3*, *51*(3), 431–444. https://doi.org/10.1038/s41588-019-0344-8

Heins, M., Achterhof, R., Collip, D., Viechtbauer, W., Kirtley, O. J., Gunther, N., … Myin-Germeys, I. (2019). Social functioning and subclinical psychosis in adolescence: a longitudinal general adolescent population study. *Acta Psychiatrica Scandinavica*, *140*(3). https://doi.org/10.1111/acps.13069

Hemani, G., Tilling, K., & Davey Smith, G. (2017). Orienting the causal relationship between imprecisely measured traits using GWAS summary data. *PLOS Genetics*, *13*(11), e1007081. https://doi.org/10.1371/JOURNAL.PGEN.1007081

Howard, D. M., Adams, M. J., Clarke, T. K., Hafferty, J. D., Gibson, J., Shirali, M., … McIntosh, A. M. (2019). Genome-wide meta-analysis of depression identifies 102 independent variants and highlights the importance of the prefrontal brain regions. *Nature Neuroscience*, *22*(3), 343–352. https://doi.org/10.1038/S41593-018-0326-7

Hu, L. T., & Bentler, P. M. (2009). Cutoff criteria for fit indexes in covariance structure analysis: Conventional criteria versus new alternatives. *Https://Doi.Org/10.1080/10705519909540118*, *6*(1), 1–55. https://doi.org/10.1080/10705519909540118

Ingram, I., Kelly, P. J., Deane, F. P., Baker, A. L., Goh, M. C. W., Raftery, D. K., & Dingle, G. A. (2020). Loneliness among people with substance use problems: A narrative systematic review. *Drug and Alcohol Review*, *39*(5), 447–483. https://doi.org/10.1111/DAR.13064

Johnson, E. C., Demontis, D., Thorgeirsson, T. E., Walters, R. K., Polimanti, R., Hatoum, A. S., … Børglum, A. D. (2020). A large-scale genome-wide association study meta-analysis of cannabis use disorder. *The Lancet Psychiatry*, *7*(12), 1032–1045. https://doi.org/10.1016/S2215-0366(20)30339-4

Kassambara, A., & Mundt, F. (2020). factoextra: Extract and Visualize the Results of Multivariate Data Analyses. R package version 1.0.7. Retrieved June 13, 2022, from https://CRAN.R-project.org/package=factoextra

Leathem, L. D., Currin, D. L., Montoya, A. K., & Karlsgodt, K. H. (2021). Socioemotional mechanisms of loneliness in subclinical psychosis. *Schizophrenia Research*, *238*. https://doi.org/10.1016/j.schres.2021.10.002

Li, C. H. (2016). The performance of ML, DWLS, and ULS estimation with robust corrections in structural equation models with ordinal variables. *Psychological Methods*, *21*(3), 369–387. https://doi.org/10.1037/MET0000093

Nievergelt, C. M., Maihofer, A. X., Klengel, T., Atkinson, E. G., Chen, C. Y., Choi, K. W., … Koenen, K. C. (2019). International meta-analysis of PTSD genome-wide association studies identifies sex- and ancestry-specific genetic risk loci. *Nature Communications 2019 10:1*, *10*(1), 1–16. https://doi.org/10.1038/s41467-019-12576-w

Otowa, T., Hek, K., Lee, M., Byrne, E. M., Mirza, S. S., Nivard, M. G., … Hettema, J. M. (2016). Meta-analysis of genome-wide association studies of anxiety disorders. *Molecular Psychiatry 2016 21:10*, *21*(10), 1391–1399. https://doi.org/10.1038/mp.2015.197

Pardiñas, A. F., Holmans, P., Pocklington, A. J., Escott-Price, V., Ripke, S., Carrera, N., … Walters, J. T. R. (2018). Common schizophrenia alleles are enriched in mutation-intolerant genes and in regions under strong background selection. *Nature Genetics 2018 50:3*, *50*(3), 381–389. https://doi.org/10.1038/s41588-018-0059-2

Revelle, W. (2020). *psych: Procedures for Personality and Psychological Research*. Illinois. Retrieved from https://personality-project.org/r/psych/

Rosseel, Y. (2012). lavaan: An R Package for Structural Equation Modeling. *Journal of Statistical Software*, *48*, 1–36. https://doi.org/10.18637/JSS.V048.I02

Schumann, G., Liu, C., O’Reilly, P., Gao, H., Song, P., Xu, B., … Elliott, P. (2016). KLB is associated with alcohol drinking, and its gene product β-Klotho is necessary for FGF21 regulation of alcohol preference. *Proceedings of the National Academy of Sciences of the United States of America*, *113*(50), 14372–14377. https://doi.org/10.1073/PNAS.1611243113

Stahl, E. A., Breen, G., Forstner, A. J., McQuillin, A., Ripke, S., Trubetskoy, V., … Sklar, P. (2019). Genome-wide association study identifies 30 loci associated with bipolar disorder. *Nature Genetics 2019 51:5*, *51*(5), 793–803. https://doi.org/10.1038/s41588-019-0397-8

Taiyun Wei, M., Taiyun Wei cre, A., Simko aut, V., Levy ctb, M., Xie ctb, Y., Jin ctb, Y., & Zemla ctb, J. (2017). R package “corrplot”: Visualization of a Correlation Matrix (Version 0.84). Retrieved June 13, 2022, from https://github.com/taiyun/corrplot

Tu, Y. K., Gunnell, D., & Gilthorpe, M. S. (2008). Simpson’s Paradox, Lord’s Paradox, and Suppression Effects are the same phenomenon - The reversal paradox. *Emerging Themes in Epidemiology*, *5*(1), 1–9. https://doi.org/10.1186/1742-7622-5-2/TABLES/4

Watanabe, K., Taskesen, E., van Bochoven, A., & Posthuma, D. (2017). Functional mapping and annotation of genetic associations with FUMA. *Nature Communications 2017 8:1*, *8*(1), 1–11. https://doi.org/10.1038/s41467-017-01261-5

Wootton, R. E., Richmond, R. C., Stuijfzand, B. G., Lawn, R. B., Sallis, H. M., Taylor, G. M. J., … Munafò, M. R. (2020). Evidence for causal effects of lifetime smoking on risk for depression and schizophrenia: a Mendelian randomisation study. *Psychological Medicine*, *50*(14), 2435–2443. https://doi.org/10.1017/S0033291719002678
